# Spider webs as reservoirs of culturable fungal diversity: evidence from orb-weaving *Cyclosa mulmeinensis* spider in Thai rice agroecosystems

**DOI:** 10.3897/BDJ.14.e187035

**Published:** 2026-04-20

**Authors:** Thanakron Into, Booppa Petcharad, Nattawut Boonyuen, Ruthada Chanklan, Srisuda Pannanusorn, Suchada Mongkolsamrit, Noppol Kobmoo, Salilaporn Nuankaew, Papichaya Kwanthong

**Affiliations:** 1 Department of Biotechnology, Faculty of Science and Technology, Thammasat University, Pathum Thani, Thailand Department of Biotechnology, Faculty of Science and Technology, Thammasat University Pathum Thani Thailand; 2 National Center for Genetic Engineering and Biotechnology (BIOTEC), National Science and Technology Development Agency (NSTDA), Pathum Thani, Thailand National Center for Genetic Engineering and Biotechnology (BIOTEC), National Science and Technology Development Agency (NSTDA) Pathum Thani Thailand

**Keywords:** non-destructive sampling, multilocus phylogeny, debris-decorated webs, Ascomycota, spider-fungal interactions, DNA barcoding, checklist

## Abstract

Spider webs are increasingly recognised as passive environmental collectors; however, fungi remain amongst the least explored biological components associated with spider silk, particularly when examined using culture-based and taxonomically resolved approaches. In this study, we present a proof-of-concept investigation of culturable fungal diversity associated with two-dimensional, debris-decorated orb webs, constructed by the orb-weaving spider *Cyclosa mulmeinensis* in rice agroecosystems in Thailand. Using a standardised field-to-laboratory isolation workflow combined with genus-appropriate multilocus phylogenetic analyses, decorated orb webs were sampled as individual units from rice agroecosystems in Thailand and fungi were isolated via dilution plating on potato dextrose agar supplemented with chloramphenicol. A total of 112 fungal isolates were recovered, grouped into 45 colony morphotypes and resolved into 23 taxa across six genera: *Alternaria*, *Aspergillus*, *Cladosporium*, *Fusarium*, *Penicillium* and *Talaromyces*. Taxonomic placement was inferred primarily from multilocus phylogenetic analyses, with morphological characteristics used as supporting evidence. Notably, several isolates formed well-supported lineages within *Cladosporium* and *Talaromyces* that could not be assigned to any described species, indicating the presence of potentially undescribed taxa. These findings demonstrate that spider webs can serve as a low-impact, non-destructive substrate for accessing viable fungal diversity in agricultural ecosystems. This approach enables reproducible culture-based recovery of taxonomically informative fungal lineages and highlights the potential of spider web sampling as a complementary tool for biodiversity assessment and environmental monitoring.

## Introduction

**Figure 1.**
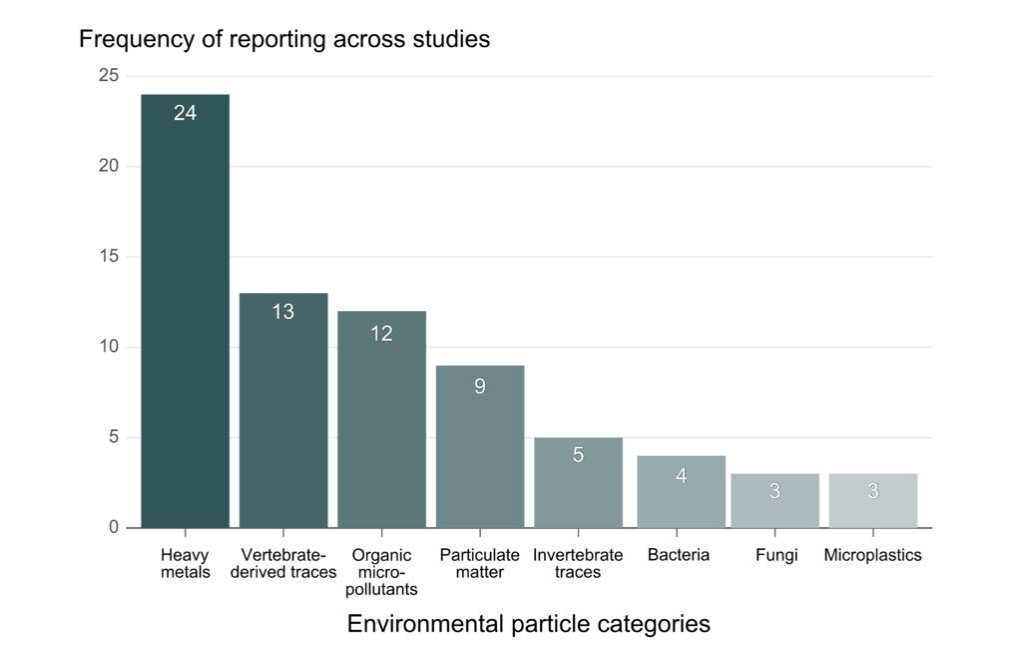
Reported categories of environmental particles detected on spider webs across published studies. Data were compiled from 33 peer-reviewed articles retrieved via Google Scholar using predefined search queries. Bars represent the number of studies in which each particle category was reported at least once. A single study may contribute to multiple categories if more than one type of material was investigated or detected. The figure illustrates relative research attention across material types rather than absolute abundance or experimental frequency.

Spider silk is a protein-based biomaterial expressed in diverse structural forms and web architectures. At the molecular level, spider silk is composed primarily of spidroin proteins and its fibre surface coated with glycoproteins and lipids that contribute to adhesion and hygroscopicity (Römer and Scheibel 2014, Opell and Stellwagen 2019, Kelly et al. 2022). These physicochemical properties allow webs to intercept, immobilise and retain airborne particles and droplets, including dust, water-borne particulates and biological materials. Consequently, spider webs have attracted increasing interest as low-cost passive sampling matrices for environmental monitoring. A synthesis of 33 experimental studies indicates that webs can capture a broad spectrum of environmental materials, but research effort has been unevenly distributed across target particle types and analytical approaches, with some biological components remaining comparatively understudied. As summarised in Fig. 1, heavy metals were the most frequently investigated materials, being assessed in all 33 studies, followed by vertebrate-derived traces (13 studies), organic micropollutants (12 studies) and particulate matter (nine studies). In contrast, biological components have been examined far less often. Only five studies assessed invertebrate traces, four reported bacteria, whereas fungi were reported in only three studies, a frequency comparable to that of microplastics (e.g. Del Fiol et al. (2007), Hubelova et al. (2021), Gregorič et al. (2022), Newton et al. (2024); Suppl. material 1). This comparative overview reveals a marked imbalance in research effort, with fungi amongst the least studied categories of materials associated with spider webs. Given the ecological importance of fungi and their ubiquity in aerial and surface-associated environments, their occurrence on spider webs remains poorly characterised — particularly using culture-based approaches coupled with taxonomically robust identification.

Fungi play fundamental roles in terrestrial ecosystems as decomposers and key drivers of nutrient cycling, thereby supporting plant productivity and shaping microbial communities (Bahram and Netherway 2021, Lofgren and Stajich 2021, Corbu et al. 2023). Although more than 120,000 fungal species have been described, this represents only a small proportion of the global fungal diversity estimated to exist (Hawksworth and Lücking 2017, Hyde et al. 2020, Hyde 2022). Fungal propagules are abundant and widely dispersed across in air, soil, vegetation and built environments, making them readily available for interception by exposed natural substrates, including spider webs (Wu et al. 2019, Vašutová et al. 2021). Despite, this direct evidence for microbial communities associated with spider-web matrices remains limited. Existing work in a small number of systems suggests that spider-produced structures can function as microhabitats: for example, studies of the social spider *Stegodyphus dumicola* show that its three-dimensional nests — enriched with accumulated organic materials — harbour diverse bacterial and fungal assemblages (Nazipi et al. 2021, Lammers et al. 2022). In orb-weaving spiders, metabarcoding and spore-based investigations of *Araneus diadematus* webs have detected fungi predominantly from Ascomycota and Basidiomycota, with genera, such as *Alternaria*, *Cladosporium* and *Fusarium*, frequently reported (Del Fiol et al. 2007, Gregorič et al. 2022). Beyond molecular detection, emerging experimental evidence indicates that webs can also function as biologically active microbial microhabitats. For example, bacteria isolated from orb webs were shown to interact with spider silk and increase its mechanical extensibility, indicating that at least some web-associated microorganisms may be active residents rather than solely passively trapped particles (Tsiareshyna et al. 2024). Nevertheless, most existing studies have relied on DNA-based detection or observational approaches, which limits recovery of viable fungal isolates and, consequently, constrains species-level taxonomic resolution, as well as downstream functional interpretation.

**Figure 2.**
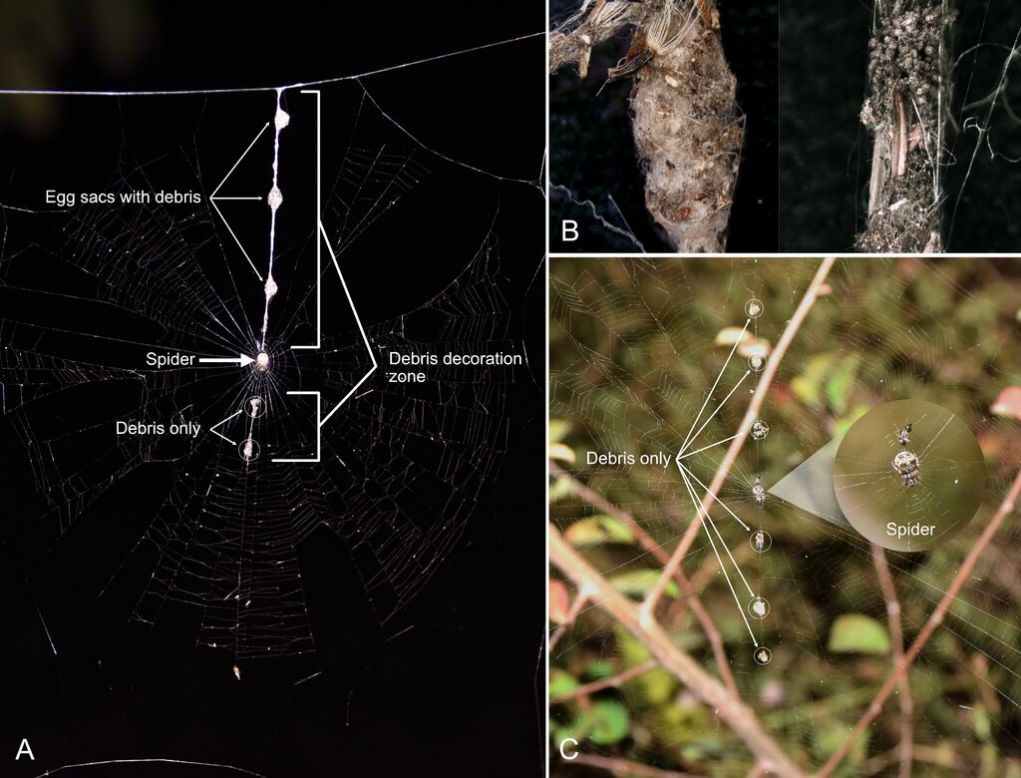
Representative orb webs of *Cyclosa mulmeinensis* illustrating web architecture and debris decoration. **A** Debris-decorated orb web with egg sacs constructed by an adult female, showing a characteristic linear debris line with egg sacs extending along a single radial thread; **B** Close-up view of the debris line, demonstrating aggregated organic materials, including plant fragments and insect remains, covering an egg sac (left) and debris only (right) incorporated into the decoration; **C** Decorated orb web with a debris line lacking egg sacs, shown to illustrate an alternative form of debris decoration without egg sacs. White circles denote debris fragments present along the debris line of the web.

The orb-weaving spiders, *Cyclosa mulmeinensis* (Thorell, 1887) (Araneae, Araneidae) represents a suitable model for investigating culturable fungi associated with spider webs. This species is widely distributed across South, East and Southeast Asia, with confirmed records from India, Bangladesh, Myanmar, China, Taiwan, Japan, Singapore and Thailand (Tanikawa 1992, World Spider Catalog 2026). *Cyclosa mulmeinensis* is a small orb-weaver (adult female body length < 6 mm) that commonly occurs in lowland and human-modified habitats, including agricultural landscapes, forest edges, mangrove forests, secondary vegetation and exposed shorelines or riverbanks (Barrion and Litsinger 1995, Liao et al. 2009, Tseng and Tso 2009). Adult females construct two-dimensional orb webs typically less than 200 mm in diameter (Liao et al. 2009). A distinctive feature of *C. mulmeinensis* is the linear debris decoration (“trashline”) positioned along a single radial thread of the web (Fig. 2A). This debris line comprises discrete clumps of organic materials, including plant fragments, insect remains, exuviae and other intercepted environmental particles (Chou et al. 2005, Blamires and Tso 2013; Fig. 2B). Such debris decorations may increase particle retention on the web and could therefore enhance the capture of microbial propagules, making decorated orb webs a promising matrix for culture-based recovery of viable fungi. The occurrence and composition of web decorations vary with developmental stage. Juvenile and subadult individuals may construct either decorated or undecorated webs and when decorations are present, they are typically smaller and lack egg sacs (Fig. 2C). In contrast, adult females commonly incorporate egg sacs into the debris line, forming a conspicuous vertical structure extending from the hub towards the upper frame of the web, although some adults may also construct undecorated webs (Liao et al. 2009, Tseng and Tso 2009). Experimental and field studies further show that debris decorations contribute to camouflage, prey attraction, predator deterrence and web persistence, thereby enhancing foraging efficiency and survival (Chou et al. 2005, Blamires and Tso 2013, Ma et al. 2020).

Despite accumulating evidence that spider webs can intercept a wide range of environmental particles, their role as substrates harbouring viable fungi remains poorly characterised. Studies reporting fungi on spider webs have largely relied on DNA-based detection or spore-based observations, which provides limited access to living isolates and, therefore, constrains species-level taxonomic resolution and downstream functional interpretation. In addition, most investigations have focused on temperate systems, leaving tropical agroecosystems — where fungal diversity and airborne propagule loads are expected to be high — comparatively understudied. From a microbiological perspective, the orb webs of *C. mulmeinensis* represent a particularly promising, yet understudied system: the combination of exposed silk surfaces and accumulated organic debris creates a heterogeneous microenvironment with the potential to intercept and retain fungal propagules present in the surrounding environment. Importantly, this study does not attempt to infer the environmental origin of web-associated fungi or to partition potential source pools (e.g. air, vegetation or soil). Instead, we adopt an exploratory, culture-based approach to demonstrate the feasibility of recovering viable fungi from *C. mulmeinensis* orb webs. Using a standardised field-to-laboratory workflow, isolates were organised into colony morphotypes and identified using genus-appropriate multilocus phylogenetic analyses. This study provides a proof-of-concept demonstration of culturable fungal recovery from spider-web substrates and establishes a reproducible framework for future ecological, taxonomic and biomonitoring applications. By expanding fungal sampling beyond conventional substrates (soil, plants and air), spider-web-based isolation may complement biodiversity surveys in human-modified landscapes and support scalable monitoring of fungal diversity in agroecosystems.

## Materials and Methods

### Collection of Cyclosa mulmeinensis spider webs

Orb webs of adult female *Cyclosa mulmeinensis* were collected from trees and surrounding vegetation along rice-field embankments at three locations in Thailand. As *C. mulmeinensis* constructs two-dimensional orb webs suspended between vegetation, intact webs were collected directly from the air without contact with surrounding surfaces using sterile 90 mm Petri dishes. Each dish was gently positioned to enclose a single web without direct hand contact with the silk, immediately sealed with parafilm, placed in a zip-lock plastic bag and transported to the laboratory in an icebox. Upon arrival, samples were stored at 4°C until further processing. Spider web sampling was conducted during two independent periods, each with distinct objectives and sampling designs.

#### Characterisation of culturable fungal richness and composition

**Figure 3.**
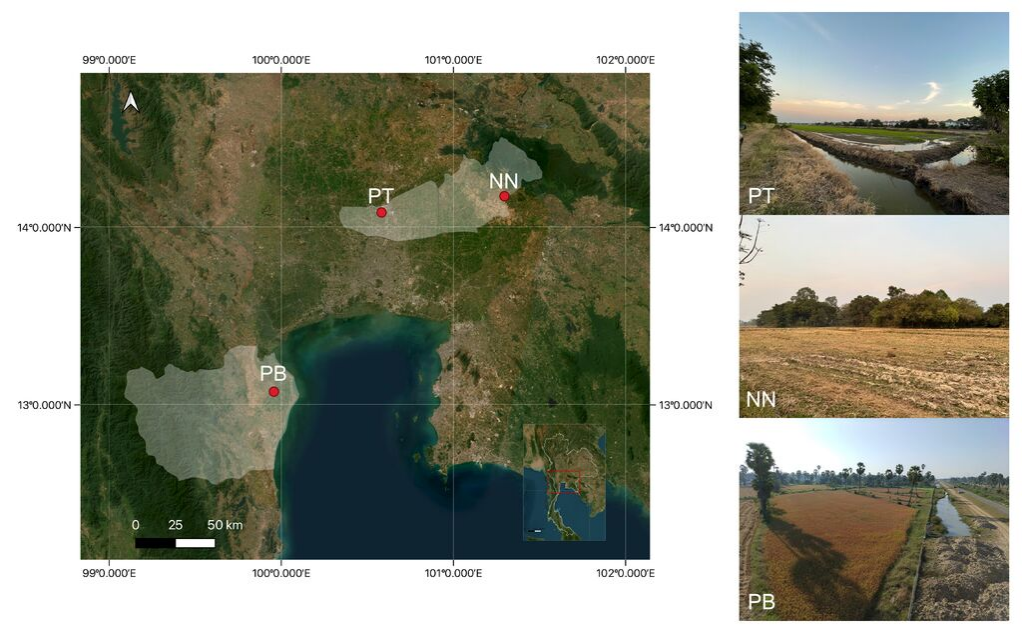
Locations of paddy fields where *Cyclosa mulmeinensis* spider webs were collected in Thailand. Sampling sites include PT (Pathum Thani Province), NN (Nakhon Nayok Province) and PB (Phetchaburi Province). These sites represent distinct geographic settings and surrounding landscape contexts and are presented to provide environmental context for sampling.

The first sampling was conducted in December 2022 to document the occurrence and taxonomic composition of culturable fungi associated with *C. mulmeinensis* orb webs, with sampling conducted across geographically distinct locations to provide environmental context. Owing to logistical constraints associated with the collection, transport and processing of intact spider webs, one debris-decorated orb web bearing egg sacs was collected per location, resulting in a total of three webs for this sampling. Sampling sites were located at: Ko Wai Sub-district, Pak Phli District, Nakhon Nayok Province (NN; 14°10'20.0"N, 101°17'48.0"E); Ban Pathum Sub-district, Sam Khok District, Pathum Thani Province (PT; 14°04'50.7''N, 100°34'58.6''E); and Ton Mamuang Sub-district, Mueang Phetchaburi District, Phetchaburi Province (PB; 13°04'15.7"N, 99°57'27.0"E) (Fig. 3). These locations differ in geographic setting and surrounding habitat characteristics and are presented here to provide environmental context only, without implying formal comparison. As sampling was conducted on different dates, storage duration at 4°C prior to processing differed amongst locations (NN: 22 days; PB: 13 days; PT: 12 days). To minimise handling-related and web-type variability, all webs collected during the first sampling were standardised to a single web category, namely debris-decorated orb webs bearing egg sacs and were processed simultaneously for fungal isolation under identical laboratory conditions. As the first sampling was designed as an exploratory assessment of culturable fungal occurrence and taxonomic composition recovered from spider webs, a single web was analysed per location. Accordingly, reported fungal richness and composition represent web-level observations rather than population-level estimates.

#### Exploratory supplementary sampling (qualitative observation only)

The second sampling represents a limited, exploratory observation intended solely to document fungal colony recovery from a small number of additional webs collected under similar field conditions. This sampling was not designed for comparative analysis and does not support any ecological inference. Sampling was conducted in January 2025 at Ko Wai Sub-district, Pak Phli District, Nakhon Nayok Province (NN). A total of three webs were collected, including debris-decorated orb webs both with and without egg sacs. Due to the limited number of samples and lack of replication, no comparison between web types was performed. All samples were stored at 4°C for approximately 12 h prior to processing and were subsequently handled using the same isolation workflow, including culture media, dilution series, incubation conditions and morphotype-to-phylogeny procedures. Observations from this sampling are presented only at the level of individual webs to illustrate variation in colony recovery and are included solely as preliminary, hypothesis-generating information for future study.

### Fungal isolation from spider webs

Web samples consisting of spider silk, either with or without visible debris decorations, were processed using a modified dilution–spread plate method. Materials retained on the web surface, including macroscopic debris in decorated webs and microscopic particulate matter in undecorated webs, were gently rinsed with 200 µl of sterile 0.9% sodium chloride (NaCl) solution to generate a suspension, hereafter referred to as the *web-associated particle suspension*. Serial dilutions (10⁻¹, 10⁻² and 10⁻³) were prepared and 100 µl aliquots from each dilution were aseptically spread on to potato dextrose agar (PDA; Difco, USA), with three replicate plates per dilution. Chloramphenicol was added to the medium at a final concentration of 250 mg/l to suppress bacterial growth. Plates were incubated at room temperature (25–28°C) for three days and were monitored daily for colony emergence. All morphologically distinct fungal colonies were identified, based on colony colour, texture and growth form, subsequently isolated and subcultured on to fresh PDA plates and incubated for an additional seven days prior to downstream analyses.

### DNA extraction and PCR amplification

Genomic DNA was extracted from all fungal isolates obtained from the first sampling. Single-colony isolates were cultivated on potato dextrose agar (PDA) at 25–28°C for two weeks to obtain sufficient mycelial biomass. Mycelial biomass was harvested using a sterile spatula and transferred into 1.5 ml microcentrifuge tubes. DNA extraction was performed using the cetyltrimethylammonium bromide (CTAB) method of Doyle and Doyle (1987), with modifications described by Mongkolsamrit et al. (2020). PCR amplifications were performed in 25 µl reaction volumes. The internal transcribed spacer (ITS) region was amplified and sequenced for all isolates. Additional loci (β-tubulin (*BenA*), Calmodulin (*CaM*), Translation elongation factor 1-alpha (*TEF1-α*), Actin (*act*) and RNA polymerase II second largest subunit (*RPB2*)) were selectively amplified for isolates requiring higher taxonomic resolution, following established genus-specific phylogenetic frameworks. Primer sets and PCR cycling conditions are summarised in Suppl. material 2 and full primer sequences and amplification profiles are provided in Suppl. material 3. PCR products were examined by electrophoresis on 1.5% agarose gels, purified and sequenced commercially by Macrogen Inc. (Seoul, Republic of Korea). Sequence chromatograms were assembled and trimmed using BioEdit v.7.1.3 (Hall 1999). All newly-generated sequences were deposited in GenBank and accession numbers, together with isolate metadata, are provided in Suppl. material 4.

### Sequence alignment and phylogenetic analyses

Consensus nucleotide sequences were compared against the NCBI database using BLASTn to identify closely-related taxa. For each genus, reference datasets were assembled by combining sequences generated in this study with sequences from type or representative strains retrieved from published studies (e.g. Bensch et al. (2010), Sandoval-Denis et al. (2016), Yilmaz et al. (2016), Houbraken et al. (2020), Nguyen et al. (2021), Visagie et al. (2024)). Multiple sequence alignments were generated using MAFFT v.7 with default parameters and manually inspected and edited in BioEdit v.7.1.3 (Hall 1999). Phylogenetic analyses were conducted using Maximum Likelihood (ML) inference implemented in IQ-TREE v.2.2.0 (Minh et al. 2020). Best-fit nucleotide substitution models were selected automatically by IQ-TREE for each dataset and branch support was assessed using ultrafast bootstrap analysis with 1,000 replicates (Kalyaanamoorthy et al. 2017). Bayesian Inference (BI) analyses were performed using MrBayes v.3.2.7 (Ronquist et al. 2012) with 10 million Markov Chain Monte Carlo (MCMC) generations, sampling every 1,000 generations. Resulting phylogenetic trees were visualised using FigTree v.1.4.4 (Rambaut 2018) and finalised using graphic editing software. Taxonomic identification of isolates was inferred, based on multilocus phylogenetic evidence. Final taxonomic assignments at the species level, species-complex level or as unresolved lineages (sp.) are provided in Suppl. material 4.

### Selection of representative isolates for morphological documentation

Following molecular identification, one representative isolate from each colony morphotype (n = 45) was selected for morphological documentation. Representative isolates correspond to the colony morphotypes and taxonomic assignments summarised in Suppl. material 4. Macroscopic colony features were examined after seven days of incubation on PDA using a stereomicroscope (Olympus SZ-PT, Tokyo, Japan). Microscopic structures were examined using a light microscope (Olympus CX31; Olympus Corporation, Japan). Diagnostic morphological traits, including conidiophores, conidia, phialides and conidial arrangement, were documented following standard mycological references (Bensch et al. 2010, Samson et al. 2010, Yilmaz et al. 2014). Permanent microscopic slides were prepared for selected morphotypes. Morphological characters were used only to support descriptive interpretation and were not employed as primary evidence for species delimitation. All representative isolates were deposited in the culture collection of the Department of Biotechnology, Faculty of Science and Technology, Thammasat University, Khlong Luang, Pathum Thani, Thailand and assigned accession codes prefixed with “BP”.

### Literature survey and data synthesis

To contextualise the ecological relevance of spider webs as substrates for fungal isolation, a literature-based data synthesis was conducted. Published experimental studies investigating materials trapped by spider webs were retrieved using Google Scholar, employing combinations of keywords including “*spider web”, “environmental monitoring”, “indicator”, “eDNA*” and “*metabarcoding*” in title and abstract searches. Only experimental studies reporting empirical detection of materials retained on spider webs were included; purely theoretical, review or non-empirical studies were excluded. Materials reported in these studies were categorised into major groups, including heavy metals, vertebrate-derived traces, organic micropollutants, particulate matter, invertebrate traces, bacteria, fungi and microplastics. The compiled dataset was cleaned, organised and summarised using RStudio (RStudio 2020). A figure was generated to illustrate comparative patterns amongst material categories and to highlight research gaps, particularly the limited number of studies reporting fungi associated with spider webs. This literature-based synthesis was conducted prior to data analysis and was intended to provide a structured contextual framework for interpreting the ecological relevance of fungal isolation from spider webs.

### Climatic data sources and contextual analysis

Long-term climatic data were compiled to provide general environmental context for the study. Secondary data on mean annual temperature and relative humidity were obtained from the Meteorological Department of Thailand for each province corresponding to the sampling sites. Data covering a 10-year period (2014–2023) prior to sample collection were used to represent prevailing regional climatic conditions (Suppl. material 5). These climatic data were not subjected to statistical analysis and were not used to infer relationships between environmental factors and fungal assemblages observed in this study. Instead, they are presented descriptively to provide background context for the broader environmental settings in which the sampled webs were collected.

## Results

### Fungal isolation and overview of taxonomic diversity

A total of 112 fungal isolates were recovered from the orb webs of *Cyclosa mulmeinensis* collected from three rice agroecosystems in Thailand. All isolates originated from debris-decorated webs bearing egg sacs collected during the first sampling. Isolates were preliminarily grouped into 45 colony morphotypes, based on macroscopic colony characteristics to facilitate downstream analyses. All quantitative summaries presented below are based on isolates recovered from a single debris-decorated web per location during the first sampling and, therefore, represent web-level observations rather than population-level estimates. Details of isolate codes, collection sites, taxonomic identification and GenBank accession numbers for sequenced loci are summarised in Suppl. material 4. Species identification was inferred primarily from multilocus phylogenetic analyses, with isolate codes explicitly linked to phylogenetic placement (Table 1; Suppl. materials 6, 7, 8, 9, 10, 11). In total, 23 fungal taxa were recovered, representing six genera and five families, including *Alternaria* (Pleosporaceae), *Aspergillus* and *Penicillium* (Aspergillaceae), *Cladosporium* (Cladosporiaceae), *Fusarium* (Nectriaceae) and *Talaromyces* (Trichocomaceae).

### Molecular identification and phylogenetic placement of fungal taxa

Taxonomic assignments presented below are based exclusively on multilocus phylogenetic analyses. Corresponding isolate codes, sampling localities and GenBank accession numbers are summarised in Suppl. material 4.

#### Aspergillus, Penicillium and Talaromyces

Phylogenetic analyses, based on concatenated ITS, *BenA* and *CaM* datasets, resolved five isolates of Aspergillus belonging to section Nigri (Suppl. material 6), including *A. aculeatinus* (three isolates: BP1-011, BP1-013, BP1-052), *A. brunneoviolaceus* (one isolate: BP1-004) and *A. niger* (one isolate: BP26-021). Within *Penicillium*, six isolates were resolved into four species, based on multilocus phylogenetic analyses across sections *Citrina*, *Charlesia* and *Lanata*-*Divaricata* (Suppl. material 7). These included *Penicillium coffeae* (one isolate: BP1-003), *P. citrinum* (three isolates: BP29-001, BP26-029, BP26-064), *P. steckii* (one isolate: BP26-040) and *P. oxalicum* (one isolate: BP1-015). Five isolates of *Talaromyces* were resolved into two described species and one putative novel lineage within section Talaromyces (Suppl. material 8). The described taxa included *Talaromyces fusiformis* (one isolate: BP1-051) and *T. alveolaris* (one isolate: BP1-057). Three isolates (BP1-048, BP1-055, BP1-058) formed a distinct and well-supported clade and were treated here as *Talaromyces* sp. 1.

#### Fusarium and Alternaria

A single isolate of *Fusarium pernambucanum* (BP26-033) was confidently resolved, based on multilocus phylogenetic analysis of concatenated *CaM*, *TEF-1α* and *RPB2* sequence datasets (Suppl. material 9).

One isolate of *Alternaria* (BP1-007) was identified to the genus level, based on ITS sequence similarity, showing 100% identity in BLAST searches. Species-level identification was not pursued, as robust species delimitation within *Alternaria* requires multilocus phylogenetic analyses beyond ITS alone, typically including loci such as *GAPDH*, *TEF-1α* and *RPB2*, together with additional diagnostic characters, in accordance with current taxonomic practice.

#### Cladosporium

Multilocus phylogenetic analyses of *Cladosporium* isolates, based on concatenated ITS, *act* and *TEF-1α* sequence datasets, resolved 26 isolates into a total of 10 species, comprising seven described species and three undescribed lineages, distributed across the *Cladosporium cladosporioides* and *C. sphaerospermum* species complexes (Suppl. materials 10, 11). Within the *C. cladosporioides* species complex, four described species were recovered: *Cladosporium angulosum* (BP26-039), *C. lagenariiforme* (BP29-012), *C. perangustum* (BP2-010) and *C. xanthochromaticum* (BP2-012, BP29-005, BP29-011, BP29-015). In addition, two well-supported undescribed lineages were detected within this complex and are treated here as *Cladosporium* sp. 1 (BP1-002, BP1-012, BP1-054, BP2-001, BP26-025, BP26-037, BP26-047, BP29-013) and *Cladosporium* sp. 2 (BP26-032). Within the *C. sphaerospermum* species complex, three described species were identified: *Cladosporium dominicanum* (BP26-068, BP29-004), *C. fusiforme* (BP2-013), and *C. velox* (BP29-008). One additional isolate formed a distinct lineage within this complex and is treated here as *Cladosporium* sp. 3 (BP26-065).

### Descriptive variation in fungal assemblages amongst individual webs

**Figure 4.**
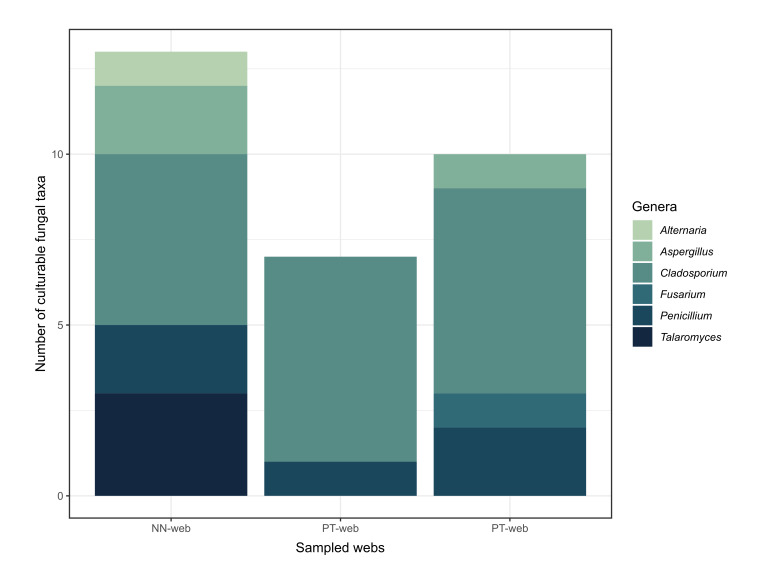
Composition of culturable fungal taxa across genera in individual sampled webs, expressed as the number of taxa recovered per web. Values represent observations from single webs and are provided for descriptive purposes only, without implying quantitative comparison.

**Figure 5.**
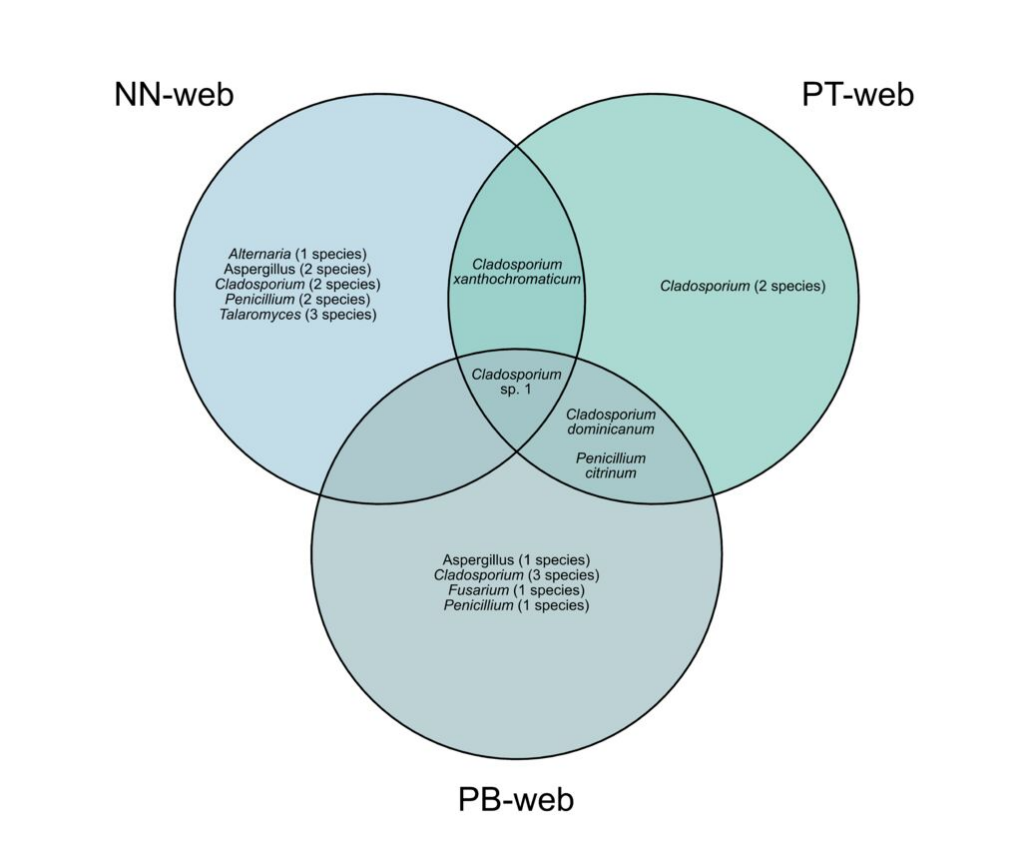
Venn diagram illustrating overlap and uniqueness of culturable fungal taxa amongst individual sampled webs.

The composition of culturable fungal taxa varied amongst the individual webs examined in this study, each being collected from a different location (Fig. 4). The web from Nakhon Nayok yielded 12 taxa across five genera, while the webs from Phetchaburi and Pathum Thani yielded nine taxa from four genera and six taxa from two genera, respectively. *Cladosporium* was the most species-rich genus observed across all sampled webs. Given that only a single web was analysed per location, these observations are presented descriptively at the level of individual samples and do not support inference regarding spatial patterns or differences amongst locations. The distribution of taxa across webs included both shared and unique occurrences. Visualisation using a Venn diagram (Fig. 5) illustrates the presence of overlapping taxa amongst webs, alongside taxa detected only in individual samples. These patterns highlight variation in culturable fungal recovery amongst webs, but should be interpreted cautiously due to the limited sample size.

### Exploratory supplementary observation of fungal recovery from debris-decorated webs (qualitative only)

In a second, exploratory sampling conducted in Nakhon Nayok Province, fungal isolation outcomes were documented from a small number of debris-decorated orb webs, including webs bearing egg sacs and webs without egg sacs. Due to the limited sample size and absence of replication, no comparative analysis was performed between web types. Variation in fungal colony abundance and growth was observed amongst individual webs (Suppl. material 12). These observations are presented descriptively at the level of individual samples and are not intended for species richness estimation, taxonomic inference or statistical evaluation. Given these limitations, this sampling should be considered preliminary and hypothesis-generating only, highlighting the need for targeted and replicated sampling to assess potential relationships between web characteristics and fungal recovery.

## Discussion

### Culturable fungi associated with Cyclosa mulmeinensis webs

This study provides a culture-based baseline of fungal biodiversity recovered from spider webs in tropical rice agroecosystems, demonstrating that spider silk is an underutilised substrate for isolating viable, taxonomically informative fungi. Using a standardised isolation workflow combined with multilocus phylogenetic analyses, we demonstrated that spider webs can harbour a diverse assemblage of viable, culturable fungi. From three independently collected webs (one web per site), we recovered 112 isolates, which were grouped into 45 colony morphotypes and resolved into 23 fungal taxa spanning six genera: *Alternaria*, *Aspergillus*, *Penicillium*, *Talaromyces*, *Cladosporium* and *Fusarium*. Previous studies documenting fungi associated with spider webs have largely relied on spore observations or molecular detection approaches, such as metabarcoding or environmental DNA analyses (Del Fiol et al. 2007, Gregorič et al. 2022). While these methods are powerful for detecting taxonomic presence, they provide limited insight into fungal viability and restrict opportunities for downstream functional or taxonomic investigation. By contrast, the culture-based approach employed here demonstrates that a substantial fraction of fungi intercepted by spider webs remains viable and can be isolated, identified and preserved for future study. This finding expands the conceptual role of spider webs from passive collectors of biological signals to substrates capable of retaining living fungal propagules. From a biodiversity and conservation perspective, non-destructive substrates such as spider webs offer a practical way to repeatedly sample fungal propagules without disturbing soil structure or plant tissues. This is particularly relevant in agroecosystems, where land-use intensity, pesticide regimes and surrounding habitat mosaics can shape airborne and surface-associated fungal pools, making standardised, low-impact sampling valuable for long-term biodiversity monitoring.

### Taxonomic composition and dominance of Cladosporium

Amongst the recovered taxa, *Cladosporium* was the most species-rich and frequently isolated genus across all sampling locations. Multilocus phylogenetic analyses resolved isolates into multiple species within the *C. cladosporioides* and *C. sphaerospermum* species complexes, including *C. angulosum*, *C. dominicanum*, *C. lagenariiforme*, *C. perangustum*, *C. velox* and *C. xanthochromaticum*, as well as several unresolved lineages. The presence of multiple unresolved lineages within intensively studied genera underscores the capacity of spider-web-based sampling to access cryptic fungal diversity that may be under-represented in conventional substrates, such as soil or plant material. The predominance of *Cladosporium* is ecologically consistent with its status as one of the most abundant and ubiquitous genera in outdoor air and on exposed substrates worldwide (Zalar et al. 2007, Bensch et al. 2015, Sandoval-Denis et al. 2016, Lee et al. 2023). Members of this genus are commonly recovered from plant surfaces, soil, insects and aquatic habitats and their frequent isolation from *C. mulmeinensis* webs supports the interpretation that spider silk functions as an efficient interceptor of airborne fungal propagules rather than as a selective growth substrate.

### Recovery of Eurotialean fungi and implications for fungal discovery

In addition to *Cladosporium*, several taxa belonging to the Eurotiales, *Aspergillus*, *Penicillium* and *Talaromyces*, were recovered and resolved using genus-appropriate multilocus phylogenetic frameworks (Yilmaz et al. 2014, Houbraken et al. 2020, Visagie et al. 2024). These genera are well known for their ecological versatility and prevalence in the air, soil and plant-associated environments, making their recovery from spider webs unsurprising. Notably, several isolates within *Talaromyces* and *Cladosporium* formed well-supported phylogenetic lineages that could not be confidently assigned to described species. This pattern suggests that spider webs may provide access to fungal diversity that remains under-represented in conventional sampling efforts. Similar conclusions have been drawn from spider web–based metabarcoding studies, which revealed unexpectedly broad assemblages of organisms originating from surrounding habitats (Gregorič et al. 2022). The recovery of potentially undescribed taxa in the present study highlights the value of spider webs as complementary substrates for fungal biodiversity exploration.

### Influence of web structure and organic debris

The observations derived from the exploratory supplementary sampling are presented solely as preliminary and hypothesis-generating and should not be interpreted as evidence of functional differences in microbial accumulation. Due to the limited sample size and absence of replication, no comparison between webs with and without egg sacs can be made. Variation in fungal colony abundance and growth was observed amongst individual debris-decorated webs. While these observations do not permit inference, it is ecologically plausible that accumulated organic debris — such as plant fragments, prey remains and soil particles — may enhance the interception and retention of fungal propagules and provide microhabitats conducive to fungal persistence. Debris decorations in *Cyclosa* webs are known to influence prey interception, camouflage, predator deterrence and web persistence (Chou et al. 2005, Blamires et al. 2010, Ma et al. 2020). Building on these established functions, it is possible that debris accumulation could also contribute to microbial retention on spider webs, although this remains to be tested under a controlled and replicated sampling design. Comparable patterns have been reported from silk-based structures of social spiders, where accumulated organic materials support diverse microbial communities, including fungi (Nazipi et al. 2021, Lammers et al. 2022). Future studies incorporating targeted sampling and replication will be necessary to evaluate these potential relationships.

### Environmental context and spatial variation

Variation in culturable fungal richness and composition was observed amongst the individual webs examined in this study, each collected from a different location. Given that only a single web was analysed per site, these observations do not support inference regarding spatial patterns or differences amongst locations. Environmental factors, such as temperature, relative humidity and wind, are well-established drivers of fungal sporulation and aerial dispersal (Jones and Harrison 2004) and may influence the pool of fungal propagules available for interception by spider webs. The sampling locations in this study differed in their broader landscape contexts, ranging from rural, vegetation-rich environments adjacent to mountainous terrain (Nakhon Nayok and Phetchaburi) to a more urbanised and industrialised setting (Pathum Thani). Such environmental heterogeneity has been associated with differences in fungal community composition in other systems (Bissett et al. 2011, Tedersoo et al. 2014, McGuire et al. 2015, Prober et al. 2015). However, the present study was not designed to evaluate environmental drivers of fungal assemblages and the observed variation cannot be attributed to specific environmental factors. These considerations are, therefore, presented as ecological context only and further studies incorporating replicated sampling across sites will be required to assess potential spatial patterns and environmental influences on fungal recovery from spider webs.

### Methodological implications and future directions

A central contribution of this study is the demonstration of a reproducible, culture-based workflow for isolating fungi from spider webs and resolving their taxonomy using multilocus phylogenetic analyses. This approach complements existing molecular detection methods by enabling the recovery of living isolates suitable for physiological, ecological and taxonomic research. Future studies incorporating seasonal replication, multiple spider species with contrasting web architectures and parallel sampling of surrounding air, vegetation and soil will be essential to disentangle the relative contributions of web structure, habitat and climate to fungal assemblages intercepted by spider silk. By establishing spider webs as viable substrates for fungal isolation, the present study provides a foundation for integrating arachnological and mycological perspectives in biodiversity assessment and environmental monitoring.

## Conclusions

This study presents a proof-of-concept demonstration that orb webs of *Cyclosa mulmeinensis* can serve as a substrate for recovering viable and taxonomically informative assemblages of culturable fungi in tropical rice agroecosystems. By applying a standardised culture-based isolation workflow combined with multilocus phylogenetic analyses, we recovered 112 fungal isolates from three independently collected webs, representing 23 taxa across six genera, with *Cladosporium* being the most species-rich group. Given the limited sample size and absence of replication, the observations presented here are descriptive at the level of individual webs and do not support inference regarding spatial patterns or environmental drivers of fungal assemblages. Nevertheless, the recovery of multiple isolates that could not be confidently assigned to described species highlights spider webs as underexplored substrates with potential value for fungal discovery. In particular, the presence of unresolved lineages within well-studied genera, such as *Cladosporium* and *Talaromyces*, suggests that spider-web-based sampling may facilitate the detection of cryptic or under-sampled fungal diversity. By establishing a reproducible workflow for isolating and identifying living fungi from spider silk, this study contributes a methodological framework for integrating mycology and arachnology. Spider webs represent accessible, low-impact substrates for culture-based fungal recovery and may serve as a complementary tool for biodiversity assessment. Further studies incorporating replicated sampling and controlled designs will be necessary to evaluate the broader ecological applicability of this approach.

## Supplementary Material

D3675219-55DA-5A75-AB9E-67BFAAFA97CA 10.3897/BDJ.14.e187035.suppl1 Supplementary material 1 Summary of published studies on spider webs as biological materials Data type Table Brief description Summary of published studies on spider webs as biological materials (n = 33), including spider species, web type, materials detected and study approach (experimental or observational). File: oo_1516238.pdf https://binary.pensoft.net/file/1516238 Thanakron Into

6E972784-10AE-527D-A5E5-3FA4D7B7B501 10.3897/BDJ.14.e187035.suppl2 Supplementary material 2 Molecular loci, primers and PCR conditions Data type Table Brief description Molecular loci, primers and PCR conditions used for multilocus phylogenetic identification of fungal isolates recovered from spider webs. File: oo_1518175.docx https://binary.pensoft.net/file/1518175 Thanakron Into

D718B7F6-F6CF-5B6D-9455-0472C37C1922 10.3897/BDJ.14.e187035.suppl3 Supplementary material 3 Primer sequences and detailed PCR conditions Data type Table Brief description Primer sequences and detailed PCR conditions used for amplification of fungal loci. File: oo_1518176.docx https://binary.pensoft.net/file/1518176 Thanakron Into

ACC3FD7C-DF35-5704-8E71-753593962F4C 10.3897/BDJ.14.e187035.suppl4 Supplementary material 4 Details of fungal isolates obtained from *Cyclosa mulmeinensis* spider webs Data type Table Brief description Details of fungal isolates obtained from *Cyclosa mulmeinensis* spider webs in Thailand, including isolate codes, collection provinces, taxonomic identification and GenBank accession numbers for sequenced loci (ITS, *BenA, CaM, act, TEF1-α and RPB2*). Taxonomic assignments are based on multilocus phylogenetic analyses and are reported at the species level, species complex level or as unresolved lineages (sp.), depending on phylogenetic resolution. File: oo_1518174.docx https://binary.pensoft.net/file/1518174 Thanakron Into

D8DDAE21-2221-51C4-A971-B4262D28297E 10.3897/BDJ.14.e187035.suppl5 Supplementary material 5 Weather data (2014–2023) Data type Image Brief description Weather data spanning ten years (2014–2023) were obtained for the three study locations, including (A) temperature, (B) relative humidity and (C) wind speed. The data were retrieved from the Information courtesy of the Meteorological Department of Thailand. File: oo_1522322.png https://binary.pensoft.net/file/1522322 Thanakron Into

87D97D0B-3912-5040-AC00-4B9830668235 10.3897/BDJ.14.e187035.suppl6 Supplementary material 6 Maximum Likelihood phylogenetic tree, representing Aspergillus section Nigri Data type Image Brief description Maximum Likelihood phylogenetic tree constructed using RAxML, representing Aspergillus section Nigri based on the combination of ITS, *BenA* and *CaM* sequence datasets. The RAxML Bootstrap support values (MLBS ≥ 70%) and the Bayesian Inference posterior probabilities (BIPP ≥ 0.95) were shown as MLBS/BIPP with *Aspergillus candidus* CBS 566.65^T^ as the outgroup. Ex-type, ex-epitype and ex-neotype strains were indicated by T, ET and NT, respectively. Strains isolated in this study were indicated in bold. File: oo_1518164.png https://binary.pensoft.net/file/1518164 Thanakron Into

A951EA19-8C65-5A7D-9A40-A753090E11E2 10.3897/BDJ.14.e187035.suppl7 Supplementary material 7 Maximum Likelihood phylogenetic tree, representing Penicillium section Citrina Data type Image Brief description Maximum Likelihood phylogenetic tree constructed using RAxML, representing Penicillium section Citrina, *Charlesia* and *Lanata*-*Divaricata*, based on the combination of ITS, *BenA* and *CaM* sequence datasets. The phylogenetic tree utilised *Penicillium chrysogenum* CBS 306.48^T^ as the outgroup for tree rooting. The RAxML Bootstrap support values (MLBS ≥ 70%) and the Bayesian Inference posterior probabilities (BIPP ≥ 0.95) were shown as MLBS/BIPP. Ex-type, ex-epitype and ex-neotype strains were indicated by T, ET and NT, respectively. Strains isolated in this study were indicated in bold. File: oo_1518168.png https://binary.pensoft.net/file/1518168 Thanakron Into

9DB7C5C0-2126-5571-BB1F-1640E354D1E3 10.3897/BDJ.14.e187035.suppl8 Supplementary material 8 Maximum Likelihood phylogenetic tree, representing Talaromyces section Talaromyces Data type Image Brief description Maximum Likelihood phylogenetic tree constructed using RAxML, representing Talaromyces section Talaromyces, based on the combination of ITS, *BenA* and *CaM* sequence datasets. The phylogenetic tree utilised *Talaromyces helicus* CBS 335.48^T^ as the outgroup for tree rooting. The RAxML Bootstrap support values (MLBS ≥ 70%) and the Bayesian Inference posterior probabilities (BIPP ≥ 0.95) were shown as MLBS/BIPP. Ex-type, ex-epitype and ex-neotype strains were indicated by T, ET and NT, respectively. Strains isolated in this study were indicated in bold. File: oo_1518169.png https://binary.pensoft.net/file/1518169 Thanakron Into

D6F51ACF-5DD6-52BC-90F2-CEF889E1CA5F 10.3897/BDJ.14.e187035.suppl9 Supplementary material 9 Maximum Likelihood phylogenetic tree, representing Fusarium incaarnatum-equiseti species complex Data type Image Brief description Maximum Likelihood phylogenetic tree constructed using RAxML, representing *Fusarium incaarnatum-equiseti* species complex (FIESC), based on the combination of *CaM*, *RPB2* and *TEF-1*α sequence datasets. The RAxML Bootstrap support values (MLBS ≥ 70%) and the Bayesian Inference posterior probabilities (BIPP ≥ 0.95) were shown as MLBS/BIPP with *Fusarium camtoceras* CBS 193.65 and *Fusarium neosemitectum* CBS189.60^T^ as the outgroups. Ex-type, ex-epitype and ex-neotype strains were indicated by T, ET and NT, respectively. Strains isolated in this study were indicated in bold File: oo_1518167.png https://binary.pensoft.net/file/1518167 Thanakron Into

E41D0422-F7CD-59FE-9901-537F8D333FA6 10.3897/BDJ.14.e187035.suppl10 Supplementary material 10 Maximum Likelihood phylogenetic tree, representing Cladosporium cladosporioides species complex Data type Image Brief description Maximum Likelihood phylogenetic tree constructed using RAxML, representing *Cladosporium cladosporioides* species complex, based on the combination of ITS, *Tef-1α* and *act* sequence datasets. The phylogenetic tree utilised *Cladosporium herbarum* CBS 121621^T^ as the outgroup for tree rooting. The RAxML Bootstrap support values (MLBS ≥ 70%) and the Bayesian Inference posterior probabilities (BIPP ≥ 0.95) were shown as MLBS/BIPP. Ex-type, ex-epitype and ex-neotype strains were indicated by T, ET and NT, respectively. Strains isolated in this study were indicated in bold. File: oo_1518165.png https://binary.pensoft.net/file/1518165 Thanakron Into

F05C1426-7B9C-508C-9E47-35D6FE2A0108 10.3897/BDJ.14.e187035.suppl11 Supplementary material 11 Maximum Likelihood phylogenetic tree, representing Cladosporium sphaerospermum species complex Data type Image Brief description Maximum Likelihood phylogenetic tree constructed using RAxML, representing *Cladosporium sphaerospermum* species complex, based on the combination of ITS, *Tef-1α* and *act* sequence datasets. The phylogenetic tree utilised *Cladosporium herbarum* CBS 121621^T^ as the outgroup for tree rooting. The RAxML Bootstrap support values (MLBS ≥ 70%) and the Bayesian Inference posterior probabilities (BIPP ≥ 0.95) were shown as MLBS/BIPP. Ex-type, ex-epitype and ex-neotype strains were indicated by T, ET and NT, respectively. Strains isolated in this study were indicated in bold. File: oo_1518166.png https://binary.pensoft.net/file/1518166 Thanakron Into

3DE9541E-8B3D-5076-ABEF-CD1EA29B477A 10.3897/BDJ.14.e187035.suppl12 Supplementary material 12 PDA plate cultures Data type Image Brief description PDA plate cultures illustrating variation in fungal colony enumeration associated with spider web structure. File: oo_1516574.png https://binary.pensoft.net/file/1516574 Thanakron Into

## Figures and Tables

**Table 1.** Details of fungal isolates obtained from *Cyclosa mulmeinensis* spider webs in Thailand including, taxonomic identification and isolated abundance. Taxonomic assignments are based on multilocus phylogenetic analyses and are reported at the species level, species complex level or as unresolved lineages (sp.), depending on phylogenetic resolution

**Family**	**Genus**	**Identified species**	**Series/Section/Complex**	**Isolate abundance**	**Clade ID**
Pleosporaceae	* Alternaria *	*Alternaria longissima*	-	1	-
Trichocomaceae	* Aspergillus *	*Aspergillus aculeatinus*	Japonici/Nigri	3	Suppl. material 6
		*Aspergillus brunneoviolaceus*		1	
		*Aspergillus niger*		1	
	* Penicillium *	*Pencillium coffeae*	Phoenicea/Charlesia	1	Suppl. material 7
		*Penicillium citrinum*	Citrina/Citrina	4	
		*Penicillium steckii*		1	
		*Penicillium oxalicum*	Oxalica/Lanata-Divaricata	1	
	* Talaromyces *	*Talaromyces alveolaris*	Talaromyces	1	Suppl. material 8
		*Talaromyces fusiformis*		1	
		*Talaromyces* sp.1		3	
Nectriaceae	* Fusarium *	*Fusarium pernambucanum*	FIESC	1	Suppl. material 9
Cladosporiaceae	* Cladosporium *	Unidentified *Cladosporium* sp.	-	5	-
		*Cladosporium angulosum*	Species complex cladosporioides	1	Suppl. material 10
		*Cladosporium lagenariiforme*		1	
		*Cladosporium perangustum*		1	
		*Cladosporium* sp. 1		8	
		*Cladosporium* sp. 2		1	
		*Cladosporium xanthochromaticum*		4	
		*Cladosporium dominicanum*	Species complex sphaerospermum	2	Suppl. material 11
		*Cladosporium fusiforme*		1	
		*Cladosporium* sp. 3		1	
		*Cladosporium velox*		1	
