# Supplementary material for: Spider webs as reservoirs of culturable fungal diversity: evidence from orb-weaving Cyclosa mulmeinensis spider in Thai rice agroecosystems
Source: Biodivers Data J. 2026 Apr 20;14:e187035. doi: 10.3897/BDJ.14.e187035 (PMC13122186; doi:10.3897/BDJ.14.e187035)
Supplement: Supplementary material 1 — Summary of published studies on spider webs as biological materials [file bdj-14-e187035-s001.pdf]

Supplementary

Table S1. Summary of published studies on spider webs as biological materials (n = 33), including spider species, web type, materials detected, and study approach (experimental or observational).

References	Measured	Family	Species	Web type	Detected methods
**Adekanmi, 2013**	Heavy metals	Unknown	Unknown	Unknown	Spider webs were washed by alcohol, digested by nitric acid and suspended in nitric acid and hydrogen peroxide. Quantitative determination of the trace metals by Atomic Absorption Spectrophotometer (AAS)
**Muhammad et al. 2014**	Heavy metals	Unknown	Unknown	Unknown	Spider webs were digested by hydrogen peroxide. Quantitative determination of the trace metals by Atomic Absorption Spectrophotometer (AAS)
**Berard et al. 2025**	Vertebrate-derived traces	Unknown	Unknown	Unknown	Molecular biological methods
**Costa et al., 2024**	Microplastics	Nephilidae	*Nephilingis cruentata*	Tangle web	Spider webs were digested by potassium hydroxide. The chemical composition was determined by Raman spectroscopy.
**Mohammed & Olagunju 2021**	Heavy metals	Unknown	Unknown	Unknown	Spider webs were washed by alcohol, digested by nitric acid and suspended in nitric acid and hydrogen peroxide. Quantitative determination of the trace metals by Atomic Absorption Spectrophotometer (AAS)
**Gregorič et al., 2022**	Bacteria	Araneidae	*Araneus diadematus*	Orb web	Molecular biological methods
	Bacteria	Linyphiidae	*Linyphia triangularis*	Tangle web	Molecular biological methods
	Fungi	Araneidae	*Araneus diadematus*	Orb web	Molecular biological methods
	Fungi	Linyphiidae	*Linyphia triangularis*	Tangle web	Molecular biological methods
	Vertebrate-derived traces	Araneidae	*Araneus diadematus*	Orb web	Molecular biological methods
	Vertebrate-derived traces	Linyphiidae	*Linyphia triangularis*	Tangle web	Molecular biological methods
**Hose et al., 2002**	Heavy metals	Desidae	*Badumna socialis*	Sheet web with funnel	Spider webs were digested by nitric acid and suspended in nitric acid and hydrogen peroxide. Heavy metals analysis was done by ion chromatograph.
	Heavy metals	Stiphidiidae	*Stiphidion facetum*	Sheet web	Spider webs were digested by nitric acid and suspended in nitric acid and hydrogen peroxide. Heavy metals analysis was done by ion chromatograph.

References	Measured	Family	Species	Web type	Detected methods
**Iordachescu et al., 2024**	Microplastics	Unknown	Unknown	Unknown	Spider webs were incubated in water and hydrogen peroxide and treated for oxidative process. FPA-μFTIR determined the chemical identity of the particles
**Joseph & Anil, 2025**	Heavy metals	Araneidae	*Cyrtophora cicatrosa*	Three-dimensional web	Heavy metals were determined by ICP-OES spectroscopy
	Heavy metals	Pholcidae	*Pholcus phalangioides*	Three-dimensional web	Heavy metals were determined by ICP-OES spectroscopy
**Aradhana et al., 2025**	Microplastics	Pholcidae	Pholcidae	Three-dimensional web	Spider webs were rinsed by hydrogen peroxide, introduced to zinc chloride to facilitate density separation. ATR-FTIR analysis were used for microplstic analysis.
	Organic micropollutants	Pholcidae	Pholcidae	Three-dimensional web	Spider webs were rinsed by hydrogen peroxide, introduced to zinc chloride to facilitate density separation. ATR-FTIR analysis were used for microplstic analysis.
**Keiser et al., 2019**	Bacteria	Eresidae	*Stegodyphus dumicola*	Three-dimensional web	Molecular biological methods
**Kim & Kim 2024**	Invertebrate traces	Unknown	Unknown	Orb web	Molecular biological methods
**Muzamil et al., 2024**	Organic micropollutants	Unknown	Unknown	Unknown	Spider webs were digested by nitric acid and hydrogen peroxide. Measurement by inductively Coupled Plasma Optical Emission Spectroscopy
**Nazipi et al., 2021**	Bacteria	Eresidae	*Stegodyphus dumicola*	Three-dimensional web	Molecular biological methods/Conventional microbiological method
	Fungi	Eresidae	*Stegodyphus dumicola*	Three-dimensional web	Molecular biological methods/Conventional microbiological method
**Newton et al., 2024**	Vertebrate-derived traces	Araneidae	*Austracantha* sp.	Orb web	Molecular biological methods
	Vertebrate-derived traces	Desidae	Badumna sp.	Irregular web	Molecular biological methods
	Vertebrate-derived traces	Nephilidae	*Nephila* sp.	Orb web	Molecular biological methods
	Vertebrate-derived traces	Nephilidae	*Trichonephila* sp.	Orb web	Molecular biological methods
	Vertebrate-derived traces	Pholcidae	*Pholcus* sp.	Three-dimensional web	Molecular biological methods
	Vertebrate-derived traces	Tetragnathidae	*Phonognatha* sp.	Orb web	Molecular biological methods
	Vertebrate-derived traces	Theridiidae	*Latrodectus* sp.	Tangle web	Molecular biological methods

References	Measured	Family	Species	Web type	Detected methods
**Rachwał et al., 2018**	Particulate matter	Agelenidae	*Agelena labyrinthica*	Sheet web	Magnetic susceptibility
	Particulate matter	Agelenidae	*Eratigena atrica*	Sheet web with funnel	Magnetic susceptibility
	Particulate matter	Linyphiidae	*Linyphia triangularis*	Tangle web	Magnetic susceptibility
	Particulate matter	Pholcidae	*Pholcus phalangioides*	Three-dimensional web	Magnetic susceptibility
**Ruiz et al., 2024**	Heavy metals	Nephilidae	*Trichonephila clavipes*	Orb web	Spider webs performed in anhydrous iron (III) chloride solution. Energy Dispersive X-ray Fluorescence Spectroscope used for heavy metal determination.
**Rutkowski et al., 2020**	Heavy metals	Agelenidae	*Agelena labyrinthica*	Sheet web	After being cleaned to remove accidental artefacts, spider webs were subjected to magnetic susceptibility (κ) measurements.
	Heavy metals	Agelenidae	*Eratigena atrica*	Sheet web with funnel	After being cleaned to remove accidental artefacts, spider webs were subjected to magnetic susceptibility (κ) measurements.
	Heavy metals	Agelenidae	*Tegenaria ferruginea*	Sheet web	After being cleaned to remove accidental artefacts, spider webs were subjected to magnetic susceptibility (κ) measurements.
	Particulate matter	Agelenidae	*Agelena labyrinthica*	Sheet web	After being cleaned to remove accidental artefacts, spider webs were subjected to magnetic susceptibility (κ) measurements.
	Particulate matter	Araneidae	*Araneus diadematus*	Orb web	After being cleaned to remove accidental artefacts, spider webs were subjected to magnetic susceptibility (κ) measurements.
**Rybak, 2015**	Organic micropollutants	Agelenidae	*Tegenaria ferruginea*		Web samples were prepared according to the paper procedure. The concentrations of elements in the webs were assessed by ICP-OES.
**Rybak, 2014**	Organic micropollutants	Agelenidae	*Tegenaria ferruginea*		PAHs content was determined with chromatograph GC-MS
	Organic micropollutants	Agelenidae	*Tegenaria silvestris*	Funnel web	PAHs content was determined with chromatograph GC-MS
	Organic micropollutants	Amaurobiidae	*Amaurobius ferox*	Irregular lace - like web	PAHs content was determined with chromatograph GC-MS
	Organic micropollutants	Theridiidae	*Theridion melanurum*	Tangle web	PAHs content was determined with chromatograph GC-MS
	Organic micropollutants	Theridiidae	*Theridion mystaceum*	Tangle web	PAHs content was determined with chromatograph GC-MS

References	Measured	Family	Species	Web type	Detected methods
**Rybak & Rutkowski, 2018**	Organic micropollutants	Agelenidae	*Tegenaria ferruginea*	Sheet web	Webs were extracted with n-hexane/acetone and concentrated on the rotary evaporator. Samples were analyzed by high resolution gas chromatograph/high resolution mass spectrometer (HRGC/HRMS).
	Organic micropollutants	Agelenidae	*Tegenaria silvestris*	Sheet web	Webs were extracted with n-hexane/acetone and concentrated on the rotary evaporator. Samples were analyzed by high resolution gas chromatograph/high resolution mass spectrometer (HRGC/HRMS).
**Rybak et al., 2015**	Heavy metals	Agelenidae	Agelenidae	Funnel web	Spider webs were digested with nitro-hydrochloric acid, after digestion was concentrated in water. ICP-OES method was used for heavy metal analysis.
**Samu et al., 2004**	Organic micropollutants	Dictynidae	*Brigittea vicina*		Small samples of spider webs were determined to organic micropollutants assessment by spectrophotometry and X-ray diffractometry.
**Sabriya Ann Seid, 2025**	Invertebrate traces	Unknown	Unknown	Unknown	Molecular biological methods
	Invertebrate traces	Unknown	Unknown	Unknown	Molecular biological methods
	Invertebrate traces	Unknown	Unknown	Unknown	Molecular biological methods
	Vertebrate-derived traces	Unknown	Unknown	Unknown	Molecular biological methods
	Vertebrate-derived traces	Unknown	Unknown	Unknown	Molecular biological methods
	Vertebrate-derived traces	Unknown	Unknown	Unknown	Molecular biological methods
**Stojanowska et al., 2020**	Heavy metals	Agelenidae	*Agelena labyrinthica*	Sheet web	The analyses of mineralized samples were determined using Flame Atomic Absorption Spectrometry (FAAS) method and Hydride Generation Atomic Absorption Spectrophotometry (HG-AAS) technique.
	Heavy metals	Agelenidae	*Eratigena atrica*	Sheet web with funnel	The analyses of mineralized samples were determined using Flame Atomic Absorption Spectrometry (FAAS) method and Hydride Generation Atomic Absorption Spectrophotometry (HG-AAS) technique.
	Heavy metals	Agelenidae	*Eratigena agrestis*	Sheet web	Spider webs were extracted according to paper protocol. The concentration of metals was determined using atomic absorption flame spectrometry (F-AAS).
	Heavy metals	Agelenidae	*Eratigena atrica*	Sheet web with funnel	Spider webs were extracted according to paper protocol. The concentration of metals was determined using atomic absorption flame spectrometry (F-AAS).

References	Measured	Family	Species	Web type	Detected methods
	Heavy metals	Desidae	*Badumna socialis*	Sheet web with funnel	Spider webs were extracted according to paper protocol. The concentration of metals was determined using atomic absorption flame spectrometry (F-AAS).
	Heavy metals	Stiphidiidae	*Stiphidion facetum*	Sheet web	Spider webs were extracted according to paper protocol. The concentration of metals was determined using atomic absorption flame spectrometry (F-AAS).
**Sun et al., 2025**	Invertebrate traces	Tetragnathidae	*Tetragnatha keyserlingi*	Orb web	Molecular biological methods
**Taher et al., 2023**	Organic micropollutants	Pholcidae	Pholcidae	Three-dimensional web	Spider webs were extracted according to paper protocol. The polycyclic aromatic hydrocarbons were examined by gas chromatography.
**Tahir et al., 2018**	Heavy metals	Pholcidae	Pholcidae	Three-dimensional web	Spider webs were extracted according to paper protocol. Atomic absorption spectrometry (AAS) was used for the analysis of metal pollution content.
**Trzyna et al., 2022**	Heavy metals	Agelenidae	*Eratigena atrica*	Sheet web with funnel	Spider webs were extracted according to paper protocol. PM deposited on threads of spider webs was analyzed with the use of SEM-EDX and ICP-MS analysis.
	Heavy metals	Linyphiidae	*Linyphia triangularis*	Tangle web	Spider webs were extracted according to paper protocol. PM deposited on threads of spider webs was analyzed with the use of SEM-EDX and ICP-MS analysis.
**Trzyna et al., 2023**	Particulate matter	Agelenidae	*Eratigena agrestis*	Sheet web	Spider webs were extracted using nitric acid and hydrogen peroxide. The inductively coupled plasma - mass spectrometry (ICP-MS) and inductively coupled plasma - optical emission spectrometry (ICP-OES) was used for determined particle elements.
	Particulate matter	Agelenidae	*Eratigena atrica*	Sheet web with funnel	Spider webs were extracted using nitric acid and hydrogen peroxide. The inductively coupled plasma - mass spectrometry (ICP-MS) and inductively coupled plasma - optical emission spectrometry (ICP-OES) was used for determined particle elements.
**van Laaten et al., 2020**	Particulate matter	Araneidae	Araneidae	Mostly orb web	Spider webs were digested following the paper protocol. Mass fractions of heavy metals were analyzed by inductively coupled plasma - mass spectrometry (ICP-MS) and inductively coupled plasma - optical emission spectrometry (ICP-OES).
**Xiao-li et al., 2006**	Heavy metals	Araneidae	*Araneus ventricosus*	Orb web	Spider webs were digested in nitric acid and hydrogen peroxide. Metal concentrations were determined by flame atomic absorption spectrometry.

References	Measured	Family	Species	Web type	Detected methods
	Heavy metals	Theridiidae	*Parasteatoda tepidariorum*	Tangle web	Spider webs were digested in nitric acid and hydrogen peroxide. Metal concentrations were determined by flame atomic absorption spectrometry.
**Yalwa & Kabo 2015**	Heavy metals	Theridiidae	*Parasteatoda tepidariorum*	Tangle web	Spider webs were digested in nitric acid and hydrogen peroxide. Metal concentrations were determined by flame atomic absorption spectrometry.
