# Supplementary material for: Spider webs as reservoirs of culturable fungal diversity: evidence from orb-weaving Cyclosa mulmeinensis spider in Thai rice agroecosystems
Source: Biodivers Data J. 2026 Apr 20;14:e187035. doi: 10.3897/BDJ.14.e187035 (PMC13122186; doi:10.3897/BDJ.14.e187035)
Supplement: Supplementary material 2 — Molecular loci, primers and PCR conditions [file bdj-14-e187035-s002.docx]

**Supplementary**

**Table S2.** Molecular loci, primers, and PCR conditions used for multilocus phylogenetic identification of fungal isolates recovered from spider webs.

| Molecular Locus | Genera | Primer Name | Amplification Profile | | | Reference |
| --- | --- | --- | --- | --- | --- | --- |
|  |  |  | Denature | Repeat Step | Extension |  |
| ITS | All genera | ITS4/ITS5 | 95 °C (1 min) | 34 cycles, 95 °C (1 min), 55 °C (2 min), 72 °C (2.30 min) | 72 °C (10 min) | (White, 1990) |
| β-tubulin (BenA) | *Aspergillus* *Penicillium* *Talaromyces* | Bt2a/Bt2b | 94 °C (3 min) | 34 cycles, 94 °C (1 min), 58/60 °C (1.30 min), 72 °C (2 min) | 72 °C (8 min) | (Glass & Donaldson, 1995) |
| Calmodulin (CaM) | *Aspergillus* *Fusarium* *Penicillium* *Talaromyces* | cmd5/cmd6 | 95 °C (1 min) | 30 cycles, 95 °C (20 s), 56 °C (15 s), 60 °C (1.15 min) | 60 °C (1.15 min) | (Hong et al., 2006) |
|  |  | CL1/CL2A | 94 °C (10 min) | 35 cycles, 94 °C (30 s), 55 °C (30 s), 72 °C (1 min) | 72 °C (7 min) | (O’Donnell et al., 2000) |
| Actin (act) | *Cladosporium* | 512F/783R | 95 °C (1 min) | 30 cycles, 95 °C (20 s), 57 °C (15 s), 60 °C (1.15 min) | 60 °C (1.15 min) | Carbone & Kohn (1999) |
| Translation elongation factor 1-alpha (TEF1-α) | *Cladosporium Fusarium* | 728F/986R | 94 °C (3 min) | 34 cycles, 94 °C (1 min), 50/54 °C (1 min), 72 °C (1.30 min) | 72 °C (8 min) | Carbone & Kohn (1999) |
| RNA polymerase II second largest subunit (RPB2) | *Fusarium* | 5F2/7CR | 95 °C (5 min) | 34 cycles, 94 °C (1 min), 58/60 °C (1.30 min), 72 °C (1.30 min) | 72 °C (10 min) | (Liu et al., 1999) |
