# Supplementary material for: Spider webs as reservoirs of culturable fungal diversity: evidence from orb-weaving Cyclosa mulmeinensis spider in Thai rice agroecosystems
Source: Biodivers Data J. 2026 Apr 20;14:e187035. doi: 10.3897/BDJ.14.e187035 (PMC13122186; doi:10.3897/BDJ.14.e187035)
Supplement: Supplementary material 3 — Primer sequences and detailed PCR conditions [file bdj-14-e187035-s003.docx]

**Supplementary**

**Table S3.** Primer sequences and detailed PCR conditions used for amplification of fungal loci.

| Gene | Primer | Primer sequences 5’ to 3’ | Orientation | Reference |
| --- | --- | --- | --- | --- |
| β-tubulin (BenA) | T10 | ACGATAGGTTCACCTCCAGAC | forward | O'Donnell & Cigelnik (1997) |
|  | Bt2a | GGTAACCAAATCGGTGCTGCTTTC | forward | Glass & Donaldson (1995) |
|  | Bt2b | ACCCTCAGTGTAGTGACCCTTGGC | reverse | Glass & Donaldson (1995) |
| Calmodulin (CaM) | cmd5 | CCGAGTACAAGGAGGCCTTC | forward | Hong *et al.* (2006) |
|  | cmd6 | CCGATAGAGGTCATAACGTGG | reverse | Hong *et al.* (2006) |
|  | CL1 | GARTWCAAGGAGGCCTTCTC | forward | O'Donnell *et al.* (2000) |
|  | CL2A | TTTTTGCATCATGAGTTGGAC | reverse | O'Donnell *et al.* (2000) |
| Translation elongation factor 1-alpha (TEF1-α) | 728F | CATCGAGAAGTTCGAGAAGG | forward | Carbone & Kohn (1999) |
|  | 986R | TACTTGAAGGAACCCTTACC | reverse | Carbone & Kohn (1999) |
|  | EF1 | ATGGGTAAGGAGGACAAGAC | forward | O’Donnell et al. (1998) |
|  | EF2 | GGARGTACCAGTSATCATGTT | reverse | O’Donnell et al. (1998) |
| Actin (act) | 512F | ATGTGCAAGGCCGGTTTCGC | forward | Carbone & Kohn (1999) |
|  | 783R | TACGAGTCCTTCTGGCCCAT | reverse | Carbone & Kohn (1999) |
| RNA polymerase II second largest subunit (RPB2) | 5F | GAYGAYMGWGATCAYTTYGG | forward | Liu et al. (1999) |
|  | 5F2 | GGGGWGAYCAGAAGAAGGC | forward | Sung et al. (2007) |
|  | 7CR | CCCATRGCTTGYTTRCCCAT | reverse | Liu et al. (1999) |
