# Supplementary material for: Spider webs as reservoirs of culturable fungal diversity: evidence from orb-weaving Cyclosa mulmeinensis spider in Thai rice agroecosystems
Source: Biodivers Data J. 2026 Apr 20;14:e187035. doi: 10.3897/BDJ.14.e187035 (PMC13122186; doi:10.3897/BDJ.14.e187035)
Supplement: Supplementary material 4 — Details of fungal isolates obtained from Cyclosa mulmeinensis spider webs [file bdj-14-e187035-s004.docx]

**Supplementary**

**Table S4.** Details of fungal isolates obtained from *Cyclosa mulmeinensis* spider webs in Thailand, including isolate codes, collection provinces, taxonomic identification, and GenBank accession numbers for sequenced loci (ITS, *BenA, CaM, act, TEF1-α and RPB2*).

Taxonomic assignments are based on multilocus phylogenetic analyses and are reported at the species level, species complex level, or as unresolved lineages (sp.), depending on phylogenetic resolution.

***Aspergillus*, *Penicillium* and *Talaromyces***

| Genus | Section | Series | species | Strain/isolate | ITS | *BenA* | *CaM* |
| --- | --- | --- | --- | --- | --- | --- | --- |
| Aspergillus | Nigri | Carbonarii | Aspergillus carbonarius | CBS 111.26^T^ | EF661204 | GU296700 | EF661167 |
| Aspergillus | Nigri | Carbonarii | Aspergillus ibericus | ITEM 4776^T^ | EF661200 | AM419748 | AJ971805 |
| Aspergillus | Nigri | Carbonarii | Aspergillus sclerotiicarbonarius | CBS 121057^T^ | EU159216 | EU159229 | EU159235 |
| Aspergillus | Nigri | Carbonarii | Aspergillus sclerotioniger | CBS 115572^T^ | DQ900606 | AY819996 | EU163271 |
| Aspergillus | Nigri | Heteromorphi | Aspergillus ellipticus | CBS 482.65^T^ | EF661221 | FJ629279 | AM117809 |
| Aspergillus | Nigri | Heteromorphi | Aspergillus heteromorphus | CBS 117.55 ^T^ | EU821305 | FJ629284 | AM421461 |
| Aspergillus | Nigri | Homomorphi | Aspergillus homomorphus | CBS 101889^T^ | EF166063 | AY820015 | FN594549 |
| Aspergillus | Nigri | Japonici | Aspergillus aculeatinus | CBS 121062 | EU159214 | EU159219 | EU159245 |
| Aspergillus | Nigri | Japonici | Aspergillus aculeatinus | CBS 121060^T^ | EU159211 | EU159220 | EU159241 |
| Aspergillus | Nigri | Japonici | Aspergillus aculeatinus | CBS 121874 | EU159208 | EU159221 | EU159240 |
| Aspergillus | Nigri | Japonici | Aspergillus aculeatinus | 6Si | - | MH063940 | MH063955 |
| Aspergillus | Nigri | Japonici | Aspergillus aculeatinus | IHEM 20714 | - | MH614576 | MH644882 |
| Aspergillus | Nigri | Japonici | Aspergillus aculeatus | CBS 172.66T | NR_111412 | HE577806 | EF661148 |
| Aspergillus | Nigri | Japonici | Aspergillus brunneoviolaceus | CBS 621.78^T^ = NRRL 4912 | AJ280003 | EF661105 | EF661147 |
| Aspergillus | Nigri | Japonici | Aspergillus brunneoviolaceus | ITEM 14784 | - | HE984411 | HE984426 |
| Aspergillus | Nigri | Japonici | Aspergillus brunneoviolaceus | PPRI 7517 | - | MK451182 | MK451328 |
| Aspergillus | Nigri | Japonici | Aspergillus brunneoviolaceus | CBS 313.89 | FJ491680 | FJ491688 | FJ491695 |
| Aspergillus | Nigri | Japonici | Aspergillus brunneoviolaceus | CBS 119.49 | FJ491679 | FJ491689 | FJ491701 |
| Aspergillus | Nigri | Japonici | Aspergillus floridensis | NRRL 62478^T^ = ITEM 14783 | - | HE984412 | HE984429 |
| Aspergillus | Nigri | Japonici | Aspergillus hydei | KUMCC 18-0196^T^ | MT152332 | MT161679 | MT178247 |
| Aspergillus | Nigri | Japonici | Aspergillus indologenus | CBS 114.80^T^ | AJ280005 | AY585539 | AM419750 |
| Aspergillus | Nigri | Japonici | Aspergillus japonicus | CBS 114.51^T^ | AJ279985 | AY585542 | AJ964875 |
| Aspergillus | Nigri | Japonici | Aspergillus labruscus | ITAL 22.223^T^ | KU708544 | KT986014 | KT986008 |
| Aspergillus | Nigri | Japonici | Aspergillus oxumiae | CCDCA 11546 T | MN431160 | - | MN531842 |
| Aspergillus | Nigri | Japonici | Aspergillus saccharolyticus | CBS 127449 ^T^ | NR_135441 | HM853553 | HM853554 |
| Aspergillus | Nigri | Japonici | Aspergillus serratalhadensis | URM 91189^T^ | MH169127 | LT993222 | LT993223 |
| Aspergillus | Nigri | Japonici | Aspergillus trinidadensis | NRRL 62479^T^ | - | HE984420 | HE984434 |
| Aspergillus | Nigri | Japonici | Aspergillus uvarum | CBS 127591^T^ | AM745757 | AM745751 | AM745755 |
| Aspergillus | Nigri | Nigri | Aspergillus brasiliensis | CBS 101740^T^ | MH862749 | AY820006 | AM295175 |
| Aspergillus | Nigri | Nigri | Aspergillus eucalypticola | CBS 122712^T^ | OL711732 | EU482435 | EU482433 |
| Aspergillus | Nigri | Nigri | Aspergillus lacticoffeatus | CBS 101883^T^ | DQ900604 | AY819998 | FN594552 |
| Aspergillus | Nigri | Nigri | Aspergillus luchuensis | CBS 205.80^T^ | JX500081 | JX500062 | JX500071 |
| Aspergillus | Nigri | Nigri | Aspergillus niger | CBS 554.65^T^ = NRRL 326 | FJ629337 | EF661089 | EF661154 |
| Aspergillus | Nigri | Nigri | Aspergillus niger | NRRL 341 | EF661187 | EF661090 | EF661155 |
| Aspergillus | Nigri | Nigri | Aspergillus niger | PPRI 7182 = CMV005A4 | - | MK451020 | MK451460 |
| Aspergillus | Nigri | Nigri | Aspergillus niger | PPRI 8734 | - | MK451032 | MK451464 |
| Aspergillus | Nigri | Nigri | Aspergillus pseudotubingensis | SDBR-CMUO2^T^ | MK457204 | MK457206 | MK457205 |
| Aspergillus | Nigri | Nigri | Aspergillus tubingensis | NRRL 4875 T | EF661193 | EF661086 | EF661151 |
| Aspergillus | Nigri | Nigri | Aspergillus tubingensis | PPRI 7393 | - | MK451021 | MK451542 |
| Aspergillus | Nigri | Nigri | Aspergillus tubingensis | DTO265-F4 | - | KU711854 | KU681057 |
| Aspergillus | Petersoniorum | Candidi | Aspergillus candidus | CBS 566.65T = TCC 1002 = IMI 091889 = NRRL 303 | EF669592 | EU014089 | EF669550 |
| Penicillium | Charlesia | Phoenicea | Penicillium aspericonidium | CBS 141832 T = DTO 030-C5 | NR_190239 | MT302240 | MT302209 |
| Penicillium | Charlesia | Fellutana | Penicillium charlesii | CBS 304.48 T = ATCC 8730 = CBS 342.51 = CECT 2277 = FRR 778 = IMI 040232 = LSHBBB127 = LSHBP146 = NRRL 1887 = NRRL 778 = QM 6338 = QM 6838 | AF033400 | JX091508 | AY741727 |
| Penicillium | Charlesia | Fellutana | Penicillium charlesii | DTO 175-H7 | MT309658 | MT302242 | MT302210 |
| Penicillium | Charlesia | Indica | Penicillium chermesinum | CBS 231.81 T = NRRL 2048 | MH861332 | KJ834441 | AY741728 |
| Penicillium | Charlesia | Indica | Penicillium chermesinum | DTO 298-18 | MT309661 | MT302245 | MT302213 |
| Penicillium | Charlesia | Phoenicea | Penicillium coffeae | CBS 119387 T = IBT 27866 = NRRL 35363 | AY742702 | KJ834443 | AY741747 |
| Penicillium | Charlesia | Phoenicea | Penicillium coffeae | NRRL 35366 | AY742705 | - | AY741750 |
| Penicillium | Charlesia | Phoenicea | Penicillium coffeae | NRRL 35365 | AY742704 | - | AY741749 |
| Penicillium | Charlesia | Phoenicea | Penicillium coffeae | NRRL 35364 | AY742703 | - | AY741748 |
| Penicillium | Charlesia | Phoenicea | Penicillium coffeae | DTO 273-A7 | MT309664 | MT302248 | - |
| Penicillium | Charlesia | Phoenicea | Penicillium coffeae | DTO 418-B1 | - | MN882783 | MN882811 |
| Penicillium | Charlesia | Costaricensia | Penicillium costaricense | DAOMC 250520 T = CBS 140998 = DTO 410-E3 = KAS 2597 | NR_158828 | KT887834 | KT887795 |
| Penicillium | Charlesia | Indica | Penicillium cuddlyae | PPRI 26355 T = CMV016A6 | MK951942 | MK951835 | MK951908 |
| Penicillium | Charlesia |  | Penicillium eremophilum | FRR 3338 T | GU733341 | KY709170 | KY611931 |
| Penicillium | Charlesia | Fellutana | Penicillium fellutanum | CBS 229.81 T = CBS 326.48 = ATCC 10443 = FRR746 = IFO 5761 = IMI 039734 = IMI 039734iii = NRRL 746 = QM 7554 | AF033399 | KJ834450 | AY741753 |
| Penicillium | Charlesia | Fellutana | Penicillium fellutanum | CBS 501.73 | JN799647 | JN799645 | JN799646 |
| Penicillium | Charlesia | Fellutana | Penicillium fusiforme | CBS 250.66 T = DTO 035-D7 | MT309668 | MT302253 | MT302220 |
| Penicillium | Charlesia | Indica | Penicillium indicum | CBS 115.63(Isotype) = NRRL3387 = ATCC 18324 = FRR 3387 = IFO 31744 = IMI 166620 | AY742699 | EU427263 | AY741744 |
| Penicillium | Charlesia | Indica | Penicillium indicum | 179.81 (type of Penicillium gerundense) | EU427291 | EU427264 | EU427283 |
| Penicillium | Charlesia | Indica | Penicillium jeongsukae | CNUFC JSK815 T | MK450725 | MK951835 | MK951908 |
| Penicillium | Charlesia | Phoenicea | Penicillium longiconidiophorum | CBS 141831 T = DTO 088-C1 | MT309669 | MT302254 | MT302221 |
| Penicillium | Charlesia | Phoenicea | Penicillium longiconidiophorum | DTO 092-C6 | MT309670 | MT302255 | MT302222 |
| Penicillium | Charlesia | Indica | Penicillium lunae | PPRI 25881 T = CMV006E6 | MK450725 | MK451088 | MK451660 |
| Penicillium | Charlesia | Phoenicea | Penicillium phoeniceum | CBS 249.32 T = ATCC 10481 = IJFM 5122 = IMI 040585 = NRRL 2070 = QM7608 = VKMF-321 | KC411711 | KJ834483 | AY741729 |
| Penicillium | Charlesia | Phoenicea | Penicillium phoeniceum | DTO 259-B9 | MT309671 | MT302256 | MT302223 |
| Penicillium | Charlesia |  | Penicillium vietnamense | DW14M | MT102836 | MT230561 | ON209438 |
| Penicillium | Citrina | Westlingiorum | Penicillium acidogenicum | CGMCC3.25421T = CC-1 | OR512884 | OR531524 | OR538539 |
| Penicillium | Citrina | Euglauca | Penicillium anatolicum | CBS 479.66 = IBT 30764 (ex-type) | AF033425 | JN606849 | JN606571 |
| Penicillium | Citrina | Westlingiorum | Penicillium aquadulcis | CNUFC JT1301 T | OK356194 | OK105100 | OK105102 |
| Penicillium | Citrina | Euglauca | Penicillium argentinense | CBS 130371 = IBT 30761 (ex-type) | JN831361 | JN606815 | JN606549 |
| Penicillium | Citrina | Westlingiorum | Penicillium atrofulvum | CBS 109.66 = DTO 31B2 = FRR 799 = IBT 30032 = IBT 29667 (ex-type) | JN617663 | JN606677 | JN606387 |
| Penicillium | Citrina | Westlingiorum | Penicillium aurantiacobrunneum | CBS 126228 = IBT 18753 (ex-type) | JN617670 | JN606702 | MN969238 |
| Penicillium | Citrina | Westlingiorum | Penicillium cairnsense | CBS 124325 = IBT 29042 (ex-type) | JN617669 | JN606693 | MN969240 |
| Penicillium | Citrina | Sumatraensia | Penicillium cerradense | UB23977 T | MT006126 | MT416533 | MT416534 |
| Penicillium | Citrina | Westlingiorum | Penicillium christenseniae | CBS 126236 = IBT 23355 (ex-type) | JN617674 | JN606680 | MN969243 |
| Penicillium | Citrina | Westlingiorum | Penicillium chrzaszczii | CBS 217.28 = FRR 903 = MUCL 29167 = NRRL 1741 = NRRL 903 (ex-type) | GU944603 | JN606758 | MN969244 |
| Penicillium | Citrina | Citrina | Penicillium citrinum | CBS 139.45 = ATCC 1109 = ATCC 36382 = CECT 2269 = FRR 1841 = IMI 091961 = IMI 092196 = LSHBAd 95 = LSHBP 25 = LSHBP 6 = MUCL 29781 = NRRL 1841 = NRRL 1842 (ex-type) | AF033422 | GU944545 | MN969245 |
| Penicillium | Citrina | Citrina | Penicillium citrinum | CBS 122396 | GU944574 | GU944554 | GU944636 |
| Penicillium | Citrina | Citrina | Penicillium citrinum | CBS 241. 85 | GU944563 | GU944546 | GU944641 |
| Penicillium | Citrina | Citrina | Penicillium citrinum | CBS 865.97 | GU944570 | GU944551 | GU944629 |
| Penicillium | Citrina | Citrina | Penicillium citrinum | CBS 101275 | GU944568 | GU944541 | GU944628 |
| Penicillium | Citrina | Copticolarum | Penicillium copticola | CBS 127355 = IBT 30771 (ex-type) | JN617685 | JN606817 | JN606553 |
| Penicillium | Citrina | Westlingiorum | Penicillium copticola | CBS 126995 = IBT 30681 (ex-type) | JN617691 | JN606733 | MN969249 |
| Penicillium | Citrina | Westlingiorum | Penicillium decaturense | CBS 117509 = NRRL 28152 = IBT 27117 (ex-type) | GU944604 | JN606685 | MN969252 |
| Penicillium | Citrina | Copticolarum | Penicillium dokdoense | MRC:SF:013606 (ex-type) | MG906868 | MH243037 | MH243031 |
| Penicillium | Citrina | Euglauca | Penicillium euglaucum | CBS 323.71 = IBT 30767 (ex-type) | JN617699 | JN606856 | JN606564 |
| Penicillium | Citrina | Galliaca | Penicillium gallaicum | CBS 167.81 = ATCC 42232 = IJFM 5597 (ex-type) | JN617690 | JN606837 | JN606548 |
| Penicillium | Citrina | Westlingiorum | Penicillium godlewskii | CBS 215.28 = ATCC 10449 = ATCC 48714 = FRR 2111 = IFO 7724 = IMI 040591 = MUCL 29243 = NRRL 2111 = QM 7566 = VKMF-1826 (ex-type) | JN617692 | JN606768 | MN969258 |
| Penicillium | Citrina | Citrina | Penicillium gorlenkoanum | CBS 408.69 = FRR 511 = IMI 140339 = VKMF-1079 (ex-type) | GU944581 | GU944520 | MN969259 |
| Penicillium | Citrina | Citrina | Penicillium gorlenkoanum | CBS 411.69 | GU944580 | GU944521 | GU944609 |
| Penicillium | Citrina | Gracilenta | Penicillium gracilentum | CBS 599.73 | KC411768 | KJ834453 | MN969260 |
| Penicillium | Citrina | Citrina | Penicillium hetheringtonii | CBS 122392 = IBT 29057 (ex-type) | GU944558 | GU944538 | MN969263 |
| Penicillium | Citrina | Citrina | Penicillium hetheringtonii | DTO 30H7 | GU944559 | - | GU944643 |
| Penicillium | Citrina | Citrina | Penicillium hetheringtonii | DTO 32E3 | GU944560 | - | GU944644 |
| Penicillium | Citrina | Westlingiorum | Penicillium manginii | CBS 253.31 = NRRL 2134 (ex-type) | GU944599 | JN606651 | MN969274 |
| Penicillium | Citrina | Westlingiorum | Penicillium miczynskii | CBS 220.28 = ATCC 10470 = DSM2437 = FRR 1077 = IFO 7730 = IMI 040030 = MUCL 29228 = NRRL 1077 = QM 1957 (ex-type) | GU944600 | JN606706 | MN969277 |
| Penicillium | Citrina | Westlingiorum | Penicillium neomiczynskii | CBS 126231 = IBT 23560 (ex-type) | JN617671 | JN606705 | MN969278 |
| Penicillium | Citrina | Westlingiorum | Penicillium nothofagi | CBS 130383 = IBT 23018 = DTO 76C2 (ex-type) | JN617712 | JN606732 | JN606507 |
| Penicillium | Citrina | Westlingiorum | Penicillium outeniquaense | CMW 56387 T | MT949903 | MT957405 | MT957450 |
| Penicillium | Citrina | Westlingiorum | Penicillium pancosmium | CBS 276.75 = DAOM 147467 = IBT 29991 (ex-type) | JN617660 | JN606790 | MN969284 |
| Penicillium | Citrina | Westlingiorum | Penicillium pasqualense | CBS 126330 = IBT 14235 (ex-type) | JN617676 | JN606673 | MN969286 |
| Penicillium | Citrina | Paxillorum | Penicillium paxilli | CBS 360.48 = ATCC 10480 = FRR 2008 = IMI 040226 = NRRL 2008 = QM 725 (ex-type) | GU944577 | JN606844 | JN606566 |
| Penicillium | Citrina | Sumatraensia | Penicillium qii | CS18-09 = CGMCC 3.25165 T | OQ870878 | OR051080 | OR051257 |
| Penicillium | Citrina | Westlingiorum | Penicillium quebecense | CBS 101623 = IBT 29050 (ex-type) | JN617661 | JN606700 | JN606509 |
| Penicillium | Citrina | Westlingiorum | Penicillium raphiae | CBS 126234 = IBT 22407 (ex-type) | JN617673 | JN606657 | MN969292 |
| Penicillium | Citrina | Sumatraensia | Penicillium rarum | CS15-04 = CGMCC 3.25166 T | OQ870881 | OR051083 | OR051260 |
| Penicillium | Citrina | Roseopurpurea | Penicillium roseopurpureum | CBS 226.29 = ATCC 10492 = ATHUM2895 = FRR 2064 = IMI 040573 = MUCL 28654 = MUCL 29237 = NRRL 2064 = NRRL 2064A (ex-type) | GU944605 | JN606838 | JN606556 |
| Penicillium | Citrina | Roseopurpurea | Penicillium sanguifluum | CBS 127032 = IBT 29041 (ex-type) | JN617681 | JN606819 | JN606555 |
| Penicillium | Citrina | Vascosobrinhoana | Penicillium sanjayi | NFCCI 5017 (ex-type) | MZ571358 | MZ558484 | MZ558492 |
| Penicillium | Citrina | Sumatraensia | Penicillium shanghaiense | SHL06-18 = CGMCC 3.27295 T | PP357620 | PP373071 | PP373076 |
| Penicillium | Citrina | Sheariorum | Penicillium shearii | CBS 290.48 = ATCC 10410 = IFO 6088 = IMI 039739 = IMI 039739iv = NRRL 715 = QM 1870 (ex-type) | GU944606 | JN606840 | EU644068 |
| Penicillium | Citrina | Citrina | Penicillium sizovae | CBS 413.69 = FRR 518 = IMI 140344 = VKMF-1073 (ex-type) | GU944588 | GU944535 | MN969298 |
| Penicillium | Citrina | Citrina | Penicillium sizovae | CBS 115968 | GU944585 | GU944533 | GU944621 |
| Penicillium | Citrina | Citrina | Penicillium sizovae | CBS 117184 | GU944587 | GU944534 | GU944620 |
| Penicillium | Citrina | Citrina | Penicillium steckii | CBS 260.55 = ATCC 10499 = CECT 2268 = DSM1252 = IMI 040583 = NRRL 2140 = QM 6413 (ex-type) | GU944597 | GU944522 | MN969300 |
| Penicillium | Citrina | Citrina | Penicillium steckii | CBS 122389 | GU944592 | GU944524 | GU944610 |
| Penicillium | Citrina | Citrina | Penicillium steckii | CBS 122388 | GU944591 | GU944525 | GU944613 |
| Penicillium | Citrina | Citrina | Penicillium steckii | CBS 122390 | GU944590 | GU944523 | GU944612 |
| Penicillium | Citrina | Citrina | Penicillium steckii | DTO 49G1 | GU944596 | - | GU944614 |
| Penicillium | Citrina | Citrina | Penicillium steckii | CBS 122391 | GU944593 | GU944528 | GU944615 |
| Penicillium | Citrina | Citrina | Penicillium steckii | NRRL 35625 | EF200085 | EF198551 | EF198579 |
| Penicillium | Citrina | Citrina | Penicillium steckii | CBS 325.59 | GU944594 | GU944527 | GU944617 |
| Penicillium | Citrina | Citrina | Penicillium steckii | CBS 789.70 | GU944595 | GU944529 | GU944616 |
| Penicillium | Citrina | Westlingiorum | Penicillium sucrivorum | CBS 135116 = DAOM 241042 = DTO 183E5 (ex-type) | JX140872 | JX141015 | JX141506 |
| Penicillium | Citrina | Sumatraensia | Penicillium sumatraense | CBS 281.36 = NRRL 779 = FRR 779 (ex-type) | GU944578 | JN606639 | MN969301 |
| Penicillium | Citrina | Copticolarum | Penicillium terrigenum | CBS 127354 = IBT 30769 (ex-type) | JN617684 | JN606810 | JN606583 |
| Penicillium | Citrina | Citrina | Penicillium tropicoides | CBS 122410 = IBT 29043 (ex-type) | GU944584 | GU944531 | MN969303 |
| Penicillium | Citrina | Citrina | Penicillium tropicoides | CBS 122436 | GU944583 | GU944530 | GU944623 |
| Penicillium | Citrina | Citrina | Penicillium tropicum | CBS 112584 = IBT 24580 (ex-type) | GU944582 | GU944532 | MN969304 |
| Penicillium | Citrina | Westlingiorum | Penicillium ubiquetum | CBS 126437 = IBT 22226 (ex-type) | JN617680 | JN606800 | MN969306 |
| Penicillium | Citrina | Roseopurpurea | Penicillium vaccaeorum | CBS 148.83 T | JN617689 | JN606835 | JN606543 |
| Penicillium | Citrina | Westlingiorum | Penicillium vancouverense | CBS 126323 = IBT 20700 (ex-type) | JN617675 | JN606663 | MN969307 |
| Penicillium | Citrina | Vascosobrinhoana | Penicillium vascosobrinhoanum | URM 8193 (ex-type) | NR_177474 | LR744069 | LR744063 |
| Penicillium | Citrina | Sumatraensia | Penicillium vulgatum | CS15-03 = CGMCC 3.25180 T | OQ870884 | OR051086 | OR051263 |
| Penicillium | Citrina | Westlingiorum | Penicillium waksmanii | CBS 230.28 = ATCC 10516 = FRR 777 = IFO 7737 = IMI 039746 = IMI 039746i = MUCL 29120 = NRRL 777 = QM 7681 (ex-type) | GU944602 | JN606779 | MN969310 |
| Penicillium | Citrina | Westlingiorum | Penicillium wellingtonense | CBS 130375 = IBT 23557 = DTO 76C6 (ex-type) | JN617713 | JN606670 | MN969311 |
| Penicillium | Citrina | Westlingiorum | Penicillium westlingii | CBS 231.28 = IMI 092272 (ex-type) | GU944601 | JN606718 | MN969312 |
| Penicillium | Lanata-Divaricata | Dalearum | Penicillium abidjanum | CBS 246.67 T | GU981582 | GU981650 | MN969234 |
| Penicillium | Lanata-Divaricata | Simplicissima | Penicillium alagoense | URM 8086 T | MK804503 | MK802333 | MK802336 |
| Penicillium | Lanata-Divaricata | Janthinella | Penicillium brefeldianum | CBS 235.81 T = NRRL 710 | AF033435 | GU981623 | EU021683 |
| Penicillium | Lanata-Divaricata | Janthinella | Penicillium cluniae | CBS 326.89 T | MN431386 | MN969376 | MN969246 |
| Penicillium | Lanata-Divaricata | Janthinella | Penicillium curticaule | CBS 135127 T | FJ231021 | JX091526 | JX141536 |
| Penicillium | Lanata-Divaricata | Dalearum | Penicillium daleae | CBS 211.28 T | GU981583 | GU981649 | MN969251 |
| Penicillium | Lanata-Divaricata | Oxalica | Penicillium diatomitis | CCF 3904T = CBS 140107T  = IBT 30728T | FJ430748 | HE651133 | LT970912 |
| Penicillium | Lanata-Divaricata | Oxalica | Penicillium diatomitis | CCF 3779 = IBT 30754 | HE651147 | HE651137 | - |
| Penicillium | Lanata-Divaricata | Oxalica | Penicillium diatomitis | MH285 | HE651149 | HE651139 | - |
| Penicillium | Lanata-Divaricata | Oxalica | Penicillium diatomitis | MH248 | HE651150 | HE651135 | - |
| Penicillium | Lanata-Divaricata | Simplicissima | Penicillium echinulonalgiovense | CBS 328.59 T | GU981587 | GU981631 | KX961269 |
| Penicillium | Lanata-Divaricata | Janthinella | Penicillium ehrlichii | CBS 324.48 T | GU981578 | GU981652 | MN969253 |
| Penicillium | Lanata-Divaricata | Janthinella | Penicillium elleniae | CBS 118135 T | GU981612 | GU981663 | MN969254 |
| Penicillium | Lanata-Divaricata | Rolfsiorum | Penicillium excelsum | IBT 31516 T | KR815341 | KP691061 | KR815342 |
| Penicillium | Lanata-Divaricata | Rolfsiorum | Penicillium fructuariae-cellae | CBS 145110 T | MK039434 | KU554679 | MK045337 |
| Penicillium | Lanata-Divaricata | Simplicissima | Penicillium globosum | CGMCC 3.18800 T = NN072354 | KY495014 | KY495123 | MN969330 |
| Penicillium | Lanata-Divaricata | Dalearum | Penicillium griseopurpureum | CBS 406.65 T | KF296408 | KF296467 | MN969261 |
| Penicillium | Lanata-Divaricata | Dalearum | Penicillium guaibinense | CCDCA 11512 T | MH674389 | MH674391 | MH674393 |
| Penicillium | Lanata-Divaricata | Rolfsiorum | Penicillium hainanense | CGMCC 3.18798 T = NN072329 | KY495009 | KY495118 | MN969333 |
| Penicillium | Lanata-Divaricata | Oxalica | Penicillium hepuense | AS 3.16039T | MW946994 | MZ004912 | MZ004916 |
| Penicillium | Lanata-Divaricata | Oxalica | Penicillium hepuense | AS 3.16040 | MW946995 | MZ004913 | MZ004917 |
| Penicillium | Lanata-Divaricata | Simplicissima | Penicillium infrabuccalum | CBS 140983 T = KAS 2181 | KT887856 | KT887817 | KT887778 |
| Penicillium | Lanata-Divaricata | Janthinella | Penicillium janthinellum | CBS 340.48 T | GU981585 | GU981625 | MN969268 |
| Penicillium | Lanata-Divaricata | Oxalica | Penicillium jiaozhouwanicum | AS 3.16038T | MW946993 | MZ004911 | MZ004915 |
| Penicillium | Lanata-Divaricata | Oxalica | Penicillium jiaozhouwanicum | AS 3.16027 | OM203537 | OM220087 | OM220088 |
| Penicillium | Lanata-Divaricata | Janthinella | Penicillium koreense | CBS 141338 T = KACC 47721 | KJ801939 | KM000846 | MN969317 |
| Penicillium | Lanata-Divaricata | Simplicissima | Penicillium laevigatum | CGMCC 3.18801 T = NN072364 | KY495015 | KY495124 | MN969335 |
| Penicillium | Lanata-Divaricata | Janthinella | Penicillium levitum | CBS 345.48 T | GU981607 | GU981654 | MN969270 |
| Penicillium | Lanata-Divaricata | Janthinella | Penicillium lineolatum | CBS 188.77 T | GU981579 | GU981620 | MN969272 |
| Penicillium | Lanata-Divaricata | Janthinella | Penicillium malacosphaerulum | CBS 135120 T = CV 2855 | FJ231026 | JX091524 | JX141542 |
| Penicillium | Lanata-Divaricata | Simplicissima | Penicillium mariae-crucis | CBS 271.83 T | GU981593 | GU981630 | MN969275 |
| Penicillium | Lanata-Divaricata | Janthinella | Penicillium ortum | CBS 135669 T = CV102 | JX091427 | JX091520 | JX141551 |
| Penicillium | Lanata-Divaricata | Oxalica | Penicillium oxalicum | CBS 219.30 T = NRRL 787 | AF033438 | KF296462 | MN969283 |
| Penicillium | Lanata-Divaricata | Oxalica | Penicillium oxalicum | CCF 2062T = IJFM 3871T  = CBS 173.81T | HE651146 | LT934389 | - |
| Penicillium | Lanata-Divaricata | Oxalica | Penicillium oxalicum | CV 822 | JX091431 | JX091528 | - |
| Penicillium | Lanata-Divaricata | Oxalica | Penicillium oxalicum | CCF 3438 | HE651143 | HE651141 | - |
| Penicillium | Lanata-Divaricata | Simplicissima | Penicillium paraherquei | CBS 338.59 T | AF178511 | KF296465 | MN969285 |
| Penicillium | Lanata-Divaricata | Dalearum | Penicillium penarojense | CBS 113178 T | GU981570 | GU981646 | MN969287 |
| Penicillium | Lanata-Divaricata | Rolfsiorum | Penicillium piscarium | CBS 362.48 T | GU981600 | GU981668 | MN969288 |
| Penicillium | Lanata-Divaricata | Rolfsiorum | Penicillium pulvillorum | CBS 280.39 T = NRRL 2026 | AF178517 | GU981670 | MN969289 |
| Penicillium | Lanata-Divaricata | Janthinella | Penicillium setosum | CBS 144865 T = WSR 62 = SBSTJP01 | KT852579 | MF184995 | MH105905 |
| Penicillium | Lanata-Divaricata | Simplicissima | Penicillium simplicissimum | CBS 372.48 T | GU981588 | GU981632 | MN969297 |
| Penicillium | Lanata-Divaricata | Simplicissima | Penicillium skrjabinii | CBS 439.75 T | GU981576 | GU981626 | MN969299 |
| Penicillium | Lanata-Divaricata | Oxalica | Penicillium soosanum | CCF 3778T = CBS 140106T  = IBT 30727T | FJ430745 | FM865811 | LT970913 |
| Penicillium | Lanata-Divaricata | Oxalica | Penicillium soosanum | CCF3905 | FJ430746 | FM865812 | - |
| Penicillium | Lanata-Divaricata | Oxalica | Penicillium soosanum | CCF3776 | LT797554 | HE651132 | - |
| Penicillium | Lanata-Divaricata | Simplicissima | Penicillium spinuliferum | CGMCC 3.18807 T = NN072545 | KY495040 | KY495149 | MN969338 |
| Penicillium | Lanata-Divaricata | Rolfsiorum | Penicillium svalbardense | CBS 122416 T | GU981603 | DQ486644 | KC346338 |
| Penicillium | Lanata-Divaricata | Simplicissima | Penicillium tanzanicum | CBS 140968 T = KAS 1946 | KT887841 | KT887802 | KT887763 |
| Penicillium | Lanata-Divaricata | Janthinella | Penicillium uruguayense | CBS 143247 T = FMR 14490 | LT904729 | LT904699 | LT904698 |
| Penicillium | Lanata-Divaricata | Rolfsiorum | Penicillium vasconiae | CBS 339.79 T | GU981599 | GU981653 | MN969309 |
| Penicillium | Lanata-Divaricata | Dalearum | Penicillium zonatum | CBS 992.72 T | GU981581 | GU981651 | MN969315 |
| Talaromyces | talaromyces |  | Talaromyces aculeatus | CBS 289.48T = NRRL2129 | KF741995 | KF741929 | KF741975 |
| Talaromyces | talaromyces |  | Talaromyces adpressus | CBS 140620T = DTO 317-G4 | KU866657 | KU866844 | KU866741 |
| Talaromyces | talaromyces |  | Talaromyces alveolaris | CBS 142379T | LT558969 | LT559086 | LT795596 |
| Talaromyces | talaromyces |  | Talaromyces amestolkiae | CBS 132696T = DTO179F5 | JX315660 | JX315623 | KF741937 |
| Talaromyces | talaromyces |  | Talaromyces angelicae | KACC 46611T = CNU 100013 = KACC 46611 | KF183638 | KF183640 | KJ885259 |
| Talaromyces | talaromyces |  | Talaromyces annesophieae | CBS 142939T = DTO 377-F3 | MF574592 | MF590098 | MF590104 |
| Talaromyces | talaromyces |  | Talaromyces apiculatus | CBS 312.59T | JN899375 | KF741916 | KF741950 |
| Talaromyces | talaromyces |  | Talaromyces aspriconidius | CBS 141835 T | MN864274 | MN863343 | MN863320 |
| Talaromyces | talaromyces |  | Talaromyces atkinsoniae | BRIP 72528a T | OP059084 | OP087524 | - |
| Talaromyces | talaromyces |  | Talaromyces aurantiacus | CBS 314.59T | JN899380 | KF741917 | KF741951 |
| Talaromyces | talaromyces |  | Talaromyces aureolinus | AS 3.15864 | MK837954 | MK837938 | MK837946 |
| Talaromyces | talaromyces |  | Talaromyces australis | CBS 137102T = IBT14256 | KF741991 | KF741922 | KF741971 |
| Talaromyces | talaromyces |  | Talaromyces bannicus | AS 3.15862 T | MK837955 | MK837939 | MK837947 |
| Talaromyces | talaromyces |  | Talaromyces beijingensis | CBS 140617T = DTO 317-D8 | KU866649 | KU866837 | KU866733 |
| Talaromyces | talaromyces |  | Talaromyces brevis | CNUFC CY2268 | OR462361 | OR507570 | OR591615 |
| Talaromyces | talaromyces |  | Talaromyces cavernicola | URM 8448 T | ON862935 | OP672383 | OP290543 |
| Talaromyces | talaromyces |  | Talaromyces cnidii | KACC 46617T = CNU 100149 | KF183639 | KF183641 | KJ885266 |
| Talaromyces | talaromyces |  | Talaromyces dendriticus | CBS 660.80T | JN899339 | JX091391 | KF741965 |
| Talaromyces | talaromyces |  | Talaromyces derxii | CBS 412.89T | JN899327 | JX494306 | KF741959 |
| Talaromyces | talaromyces |  | Talaromyces dimorphus | AS3.15692T = NN072337 | KY007095 | KY007111 | KY007103 |
| Talaromyces | talaromyces |  | Talaromyces dimorphus | GXM23012 | OQ550088 | PP869306 | PQ095610 |
| Talaromyces | talaromyces |  | Talaromyces duclauxii | CBS 322.48T | JN899342 | JX091384 | KF741955 |
| Talaromyces | talaromyces |  | Talaromyces echinulatus | CNUFC HB1206T | OR462362 | OR507571 | OR608367 |
| Talaromyces | talaromyces |  | Talaromyces euchlorocarpius | PF 1203T = CBM-FA-0942 = DTO176I3 | AB176617 | KJ865733 | KJ885271 |
| Talaromyces | talaromyces |  | Talaromyces flavovirens | CBS 102801T | JN899392 | JX091376 | KF741933 |
| Talaromyces | talaromyces |  | Talaromyces flavus | CBS 310.38T | JN899360 | JX494302 | KF741949 |
| Talaromyces | talaromyces |  | Talaromyces francoae | CBS 113134T = DTO 056D9 = DTO 317-F4 | KX011510 | KX011489 | KX011501 |
| Talaromyces | talaromyces |  | Talaromyces fusiformis | CNUFC AS2-6 | OR462363 | OR507573 | OR608369 |
| Talaromyces | talaromyces |  | Talaromyces galapagensis | CBS 751.74T | JN899358 | JX091388 | KF741966 |
| Talaromyces | talaromyces |  | Talaromyces ginkgonis | CGMCC 3.20698 T | OL638158 | OL689844 | OL689846 |
| Talaromyces | talaromyces |  | Talaromyces haitouensis | AS 3.16101 T | MZ045695 | MZ054634 | MZ054637 |
| Talaromyces | talaromyces |  | Talaromyces indigoticus | CBS 100534T | JN899331 | JX494308 | KF741931 |
| Talaromyces | talaromyces |  | Talaromyces intermedius | CBS 152.65T | JN899332 | JX091387 | KJ885290 |
| Talaromyces | talaromyces |  | Talaromyces kendrickii | CBS 136666T = IBT13593 | KF741987 | KF741921 | KF741967 |
| Talaromyces | talaromyces |  | Talaromyces lentulus | AS3.15689T = NN071323 | KY007088 | KY007104 | KY007096 |
| Talaromyces | talaromyces |  | Talaromyces liani | CBS 225.66T | JN899395 | JX091380 | KJ885257 |
| Talaromyces | talaromyces |  | Talaromyces mae | AS3.15690T = NN071328 | KY007090 | KY007106 | KY007098 |
| Talaromyces | talaromyces |  | Talaromyces mangshanicus | HMAS 248733T | KX447531 | KX447530 | KX447528 |
| Talaromyces | talaromyces |  | Talaromyces marneffei | CBS 388.87T | JN899344 | JX091389 | KF741958 |
| Talaromyces | talaromyces |  | Talaromyces muroii | CNUFC HRGP2 | OR462364 | OR507572 | OR591616 |
| Talaromyces | talaromyces |  | Talaromyces mycothecae | URM 7622T | MF278326 | LT855561 | LT855564 |
| Talaromyces | talaromyces |  | Talaromyces nanjingensis | CCTCC-M-2012167 T | MW130720 | MW147759 | MW147760 |
| Talaromyces | talaromyces |  | Talaromyces neofusisporus | AS3.15415T | KP765385 | KP765381 | KP765383 |
| Talaromyces | talaromyces |  | Talaromyces oumae-annae | CBS 138208T = DTO_269E8 | KJ775720 | KJ775213 | KJ775425 |
| Talaromyces | talaromyces |  | Talaromyces panamensis | CBS 128.89T | JN899362 | HQ156948 | KF741936 |
| Talaromyces | talaromyces |  | Talaromyces penicillioides | AS 3.15822 T | MK837956 | MK837940 | MK837948 |
| Talaromyces | talaromyces |  | Talaromyces pinophilus | CBS 631.66T | JN899382 | JX091381 | KF741964 |
| Talaromyces | talaromyces |  | Talaromyces pratensis | NRRL 62170 | MH793075 | MH792948 | MH793012 |
| Talaromyces | talaromyces |  | Talaromyces purpureogenus | CBS 286.36T | JN899372 | JX315639 | KF741947 |
| Talaromyces | talaromyces |  | Talaromyces qii | AS3.15414T | KP765384 | KP765380 | KP765382 |
| Talaromyces | talaromyces |  | Talaromyces rosorhizae | GUCC 197011.1 | MZ221604 | MZ333144 | MZ333138 |
| Talaromyces | talaromyces |  | Talaromyces ruber | CNUFC U7-11C | OR462365 | OR507574 | OR591617 |
| Talaromyces | talaromyces |  | Talaromyces rubicundus | CBS 342.59T | JN899384 | JX494309 | KF741956 |
| Talaromyces | talaromyces |  | Talaromyces rufus | DTO 274-C5 | MN864273 | MN863342 | MN863319 |
| Talaromyces | talaromyces |  | Talaromyces santanderensis | HF05 T | OP082331 | OP067657 | OP067656 |
| Talaromyces | talaromyces |  | Talaromyces sayulitensis | CBS 138204T = DTO_245H1 | KJ775713 | KJ775206 | KJ775422 |
| Talaromyces | talaromyces |  | Talaromyces shilinensis | CGMCC 3.20699 T | OL638159 | OL689845 | OL689847 |
| Talaromyces | talaromyces |  | Talaromyces siamensis | CBS 475.88T | JN899385 | JX091379 | KF741960 |
| Talaromyces | talaromyces |  | Talaromyces soli | NRRL 62165T | MH793074 | MH792947 | MH793011 |
| Talaromyces | talaromyces |  | Talaromyces sparsus | AS 3.15880 T | MK837958 | MK837942 | MK837950 |
| Talaromyces | talaromyces |  | Talaromyces stellenboschensis | CBS 135665T = CV104 | JX091471 | JX091605 | JX140683 |
| Talaromyces | talaromyces |  | Talaromyces stipitatus | CBS 375.48T | JN899348 | KM111288 | KF741957 |
| Talaromyces | talaromyces |  | Talaromyces stollii | CBS 408.93T | JX315674 | JX315633 | JX315646 |
| Talaromyces | talaromyces |  | Talaromyces thailandensis | CBS 133147T | JX898041 | JX494294 | KF741940 |
| Talaromyces | talaromyces |  | Talaromyces verruculosus | DTO 264-I8T = NRRL1050 = CBS 254.56 | KF741994 | KF741928 | KF741944 |
| Talaromyces | talaromyces |  | Talaromyces viridis | CBS 114.72T | AF285782 | JX494310 | KF741935 |
| Talaromyces | talaromyces |  | Talaromyces wushanicus | CS17-05T | MZ356356 | MZ361347 | MZ361354 |
| Talaromyces | talaromyces |  | Talaromyces xishaensis | CGMCC 3.17995T | KU644580 | KU644581 | KU644582 |
| Talaromyces | talaromyces |  | Talaromyces yunnanensis | KUMCC 18-0208 T | MT152339 | MT161683 | MT178251 |
| Talaromyces | talaromyces |  | Talaromyces zhenhaiensis | AS 3.16102 T | MZ045697 | MZ054636 | MZ054639 |

***Cladosporium***

| Genus | Species complex | Species | Strain/Isolate | ITS | act | TEF1-α |
| --- | --- | --- | --- | --- | --- | --- |
| *Cladosporium* | *cladosporioides* | *Cladosporium alboflavescens* | CBS 140690T | LN834420 | LN834604 | LN834516 |
| *Cladosporium* | *cladosporioides* | *Cladosporium angulosum* | CBS 140692T | LN834425 | LN834609 | LN834521 |
| *Cladosporium* | *cladosporioides* | *Cladosporium angustisporum* | CBS 125983T | HM147995 | HM148482 | HM148236 |
| *Cladosporium* | *cladosporioides* | *Cladosporium angustiterminale* | CBS 140480T | KT600379 | KT600575 | KT600476 |
| *Cladosporium* | *cladosporioides* | *Cladosporium anthropophilum* | CBS 140685T | LN834437 | LN834621 | LN834533 |
| *Cladosporium* | *cladosporioides* | *Cladosporium arenosum* | CHFC-EA-566T | MN879328 | MN890008 | MN890011 |
| *Cladosporium* | *cladosporioides* | *Cladosporium asperulatum* | CBS 126340T | HM147998 | HM148485 | HM148239 |
| *Cladosporium* | *cladosporioides* | *Cladosporium australiense* | CBS 125984T | HM147999 | HM148486 | HM148240 |
| *Cladosporium* | *cladosporioides* | *Cladosporium austroafricanum* | CBS 140481T | KT600381 | KT600577 | KT600478 |
| *Cladosporium* | *cladosporioides* | *Cladosporium caprifimosum* | FMR 16532T | LR813198 | LR813205 | LR813210 |
| *Cladosporium* | *cladosporioides* | *Cladosporium cavernicola* | URM 8389T | MZ518829 | MZ555746 | MZ555733 |
| *Cladosporium* | *cladosporioides* | *Cladosporium chalastosporoides* | CBS 125985T | HM148001 | HM148488 | HM148242 |
| *Cladosporium* | *cladosporioides* | *Cladosporium chasmanthicola* | CBS 142612T | KY646221 | KY646224 | KY646227 |
| *Cladosporium* | *cladosporioides* | *Cladosporium chubutense* | CBS 124457T | FJ936158 | FJ936165 | FJ936161 |
| *Cladosporium* | *cladosporioides* | *Cladosporium cladosporioides* | CBS 101367 | HM148002 | HM148489 | HM148243 |
| *Cladosporium* | *cladosporioides* | *Cladosporium cladosporioides* | CBS 112388T | HM148003 | HM148490 | HM148244 |
| *Cladosporium* | *cladosporioides* | *Cladosporium colocasiae* | CBS 386-64T | HM148067 | HM148555 | HM148310 |
| *Cladosporium* | *cladosporioides* | *Cladosporium colombiae* | CBS 274-80BT | FJ936159 | FJ936166 | FJ936163 |
| *Cladosporium* | *cladosporioides* | *Cladosporium coprophilum* | FMR 16164T | LR813201 | LR813207 | LR813213 |
| *Cladosporium* | *cladosporioides* | *Cladosporium crousii* | CBS 140686T | LN834431 | LN834615 | LN834527 |
| *Cladosporium* | *cladosporioides* | *Cladosporium cucumerinum* | CBS 171-52T | HM148072 | HM148561 | HM148316 |
| *Cladosporium* | *cladosporioides* | *Cladosporium delicatulum* | CBS 126344T | HM148081 | HM148570 | HM148325 |
| *Cladosporium* | *cladosporioides* | *Cladosporium devikae* | BRIP 72278aT | MZ303808 | MZ344212 | MZ344193 |
| *Cladosporium* | *cladosporioides* | *Cladosporium endoviticola* | JZB390018T | MN654960 | MN984220 | MN984228 |
| *Cladosporium* | *cladosporioides* | *Cladosporium europaeum* | CBS 134914T | HM148056 | HM148543 | HM148298 |
| *Cladosporium* | *cladosporioides* | *Cladosporium exasperatum* | CBS 125986T | HM148090 | HM148579 | HM148334 |
| *Cladosporium* | *cladosporioides* | *Cladosporium exile* | CBS 125987T | HM148091 | HM148580 | HM148335 |
| *Cladosporium* | *cladosporioides* | *Cladosporium flabelliforme* | CBS 126345T | HM148092 | HM148581 | HM148336 |
| *Cladosporium* | *cladosporioides* | *Cladosporium flavovirens* | CBS 140462T | LN834440 | LN834624 | LN834536 |
| *Cladosporium* | *cladosporioides* | *Cladosporium funiculosum* | CBS 122129T | HM148094 | HM148583 | HM148338 |
| *Cladosporium* | *cladosporioides* | *Cladosporium fuscoviride* | FMR 16385T | LR813200 | LR813206 | LR813212 |
| *Cladosporium* | *cladosporioides* | *Cladosporium gamsianum* | CBS 125989T | HM148095 | HM148584 | HM148339 |
| *Cladosporium* | *cladosporioides* | *Cladosporium globisporum* | CBS 812-96T | HM148096 | HM148585 | HM148340 |
| *Cladosporium* | *cladosporioides* | *Cladosporium grevilleae* | CBS 114271T | JF770450 | JF770473 | JF770472 |
| *Cladosporium* | *cladosporioides* | *Cladosporium herbarum* | CBS 121621T | EF679363 | EF679516 | EF679440 |
| *Cladosporium* | *cladosporioides* | *Cladosporium hillianum* | CBS 125988T | HM148097 | HM148586 | HM148341 |
| *Cladosporium* | *cladosporioides* | *Cladosporium inversicolor* | CBS 401-80T | HM148101 | HM148590 | HM148345 |
| *Cladosporium* | *cladosporioides* | *Cladosporium ipereniae* | CBS 140483T | KT600394 | KT600589 | KT600491 |
| *Cladosporium* | *cladosporioides* | *Cladosporium iranicum* | CBS 126346T | HM148110 | HM148599 | HM148354 |
| *Cladosporium* | *cladosporioides* | *Cladosporium kenpeggii* | CBS 142613T | KY646222 | KY646225 | KY646228 |
| *Cladosporium* | *cladosporioides* | *Cladosporium lagenariiforme* | SFC20230103-M20 | OQ186116 | OQ185164 | OQ185125 |
| *Cladosporium* | *cladosporioides* | *Cladosporium lagenariiforme* | SFC20230103-M23 | OQ186119 | OQ185167 | OQ185128 |
| *Cladosporium* | *cladosporioides* | *Cladosporium lentulum* | FMR 16288T | LR813203 | LR813209 | LR813215 |
| *Cladosporium* | *cladosporioides* | *Cladosporium licheniphilum* | CBS 125990T | HM148111 | HM148600 | HM148355 |
| *Cladosporium* | *cladosporioides* | *Cladosporium longicatenatum* | CBS 140485T | KT600403 | KT600598 | KT600500 |
| *Cladosporium* | *cladosporioides* | *Cladosporium lycoperdinum* | CBS 574-78C | HM148115 | HM148604 | HM148359 |
| *Cladosporium* | *cladosporioides* | *Cladosporium macadamiae* | BRIP 72269aT | MZ303810 | MZ344214 | MZ344195 |
| *Cladosporium* | *cladosporioides* | *Cladosporium macadamiae* | COAD 3373 | - | OQ605785 | OQ605776 |
| *Cladosporium* | *cladosporioides* | *Cladosporium magnoliigena* | MFLUCC 18-1559T | MK347813 | - | MK340864 |
| *Cladosporium* | *cladosporioides* | *Cladosporium marinum* | SFC20230103-M33 | OQ186129 | OQ185177 | OQ185137 |
| *Cladosporium* | *cladosporioides* | *Cladosporium martimaltirimosum* | SFC20230103-M41 | OQ186137 | OQ185185 | OQ185145 |
| *Cladosporium* | *cladosporioides* | *Cladosporium montecillanum* | CBS 140486T | KT600406 | KT600602 | KT600504 |
| *Cladosporium* | *cladosporioides* | *Cladosporium myrtacearum* | CBS 126350T | HM148117 | HM148606 | HM148361 |
| *Cladosporium* | *cladosporioides* | *Cladosporium needhamense* | CBS 143359T | MF473142 | MF473991 | MF473570 |
| *Cladosporium* | *cladosporioides* | *Cladosporium neerlandicum* | CBS 143360T | KP701887 | KP702010 | KP701764 |
| *Cladosporium* | *cladosporioides* | *Cladosporium neopsychrotolerans* | CGMCC 3-18031T | KX938383 | KX938366 | KX938400 |
| *Cladosporium* | *cladosporioides* | *Cladosporium oxysporum* | CBS 125991 | HM148118 | HM148607 | HM148362 |
| *Cladosporium* | *cladosporioides* | *Cladosporium oxysporum* | CBS 126351 | HM148119 | HM148608 | HM148363 |
| *Cladosporium* | *cladosporioides* | *Cladosporium paracladosporioides* | CBS 171.54T | HM148120 | HM148609 | HM148364 |
| *Cladosporium* | *cladosporioides* | *Cladosporium parapenidielloides* | CBS 140487T | KT600410 | KT600606 | KT600508 |
| *Cladosporium* | *cladosporioides* | *Cladosporium passiflorae* | COAD 2135T | MH682175 | MH729795 | MH724943 |
| *Cladosporium* | *cladosporioides* | *Cladosporium perangustum* | CBS 125996T | HM148121 | HM148610 | HM148365 |
| *Cladosporium* | *cladosporioides* | *Cladosporium perangustum* | CPC 13870 | HM148142 | HM148631 | HM148386 |
| *Cladosporium* | *cladosporioides* | *Cladosporium perangustum* | SFC20230103-M02 | OQ186112 | OQ185160 | OQ185121 |
| *Cladosporium* | *cladosporioides* | *Cladosporium pernambucoense* | URM 8390T | MZ518830 | MZ555747 | MZ555734 |
| *Cladosporium* | *cladosporioides* | *Cladosporium phaenocomae* | CBS 128769T | JF499837 | JF499881 | JF499875 |
| *Cladosporium* | *cladosporioides* | *Cladosporium phyllactiniicola* | CBS 126352T | HM148150 | HM148639 | HM148394 |
| *Cladosporium* | *cladosporioides* | *Cladosporium phyllophilum* | CBS 125992T | HM148154 | HM148643 | HM148398 |
| *Cladosporium* | *cladosporioides* | *Cladosporium pini-ponderosae* | CBS 124456T | FJ936160 | FJ936167 | FJ936164 |
| *Cladosporium* | *cladosporioides* | *Cladosporium proteacearum* | BRIP 72301aT | MZ303809 | MZ344213 | MZ344194 |
| *Cladosporium* | *cladosporioides* | *Cladosporium pseudochalastosporoides* | CBS 140490T | KT600415 | KT600611 | KT600513 |
| *Cladosporium* | *cladosporioides* | *Cladosporium pseudocladosporioides* | CBS 125993T | HM148158 | HM148647 | HM148402 |
| *Cladosporium* | *cladosporioides* | *Cladosporium puris* | COAD 2494 | MK253338 | MK249981 | MK293778 |
| *Cladosporium* | *cladosporioides* | *Cladosporium rectoides* | CBS 125994T | HM148193 | HM148683 | HM148438 |
| *Cladosporium* | *cladosporioides* | *Cladosporium rubrum* | CMG 28T | MN053018 | MN066639 | MN066644 |
| *Cladosporium* | *cladosporioides* | *Cladosporium ruguloflabelliforme* | CBS 140494T | KT600458 | KT600655 | KT600557 |
| *Cladosporium* | *cladosporioides* | *Cladosporium rugulovarians* | CBS 140495T | KT600459 | KT600656 | KT600558 |
| *Cladosporium* | *cladosporioides* | *Cladosporium scabrellum* | CBS 126358T | HM148195 | HM148685 | HM148440 |
| *Cladosporium* | *cladosporioides* | *Cladosporium silenes* | CBS 109082T | EF679354 | EF679506 | EF679429 |
| *Cladosporium* | *cladosporioides* | *Cladosporium sinuatum* | CGMCC 3-18096T | KX938385 | KX938368 | KX938402 |
| *Cladosporium* | *cladosporioides* | *Cladosporium subuliforme* | CBS 126500T | HM148196 | HM148686 | HM148441 |
| *Cladosporium* | *cladosporioides* | *Cladosporium tenuissimum* | CBS 125995T | HM148197 | HM148687 | HM148442 |
| *Cladosporium* | *cladosporioides* | *Cladosporium tenuissimum* | C120302 | KJ558396 | KJ558393 | KJ558401 |
| *Cladosporium* | *cladosporioides* | *Cladosporium tenuissimum* | CPC 22398 | MF473285 | MF474135 | MF473708 |
| *Cladosporium* | *cladosporioides* | *Cladosporium tianshanense* | CGMCC 3-18033T | KX938381 | KX938364 | KX938398 |
| *Cladosporium* | *cladosporioides* | *Cladosporium uredinicola* | CPC 5390 | AY251071 | HM148712 | HM148467 |
| *Cladosporium* | *cladosporioides* | *Cladosporium uwebraunianum* | CBS 143365T | MF473306 | MF474156 | MF473729 |
| *Cladosporium* | *cladosporioides* | *Cladosporium varians* | CBS 126362T | HM148224 | HM148715 | HM148470 |
| *Cladosporium* | *cladosporioides* | *Cladosporium verrucocladosporioides* | CBS 126363T | HM148226 | HM148717 | HM148472 |
| *Cladosporium* | *cladosporioides* | *Cladosporium vicinum* | CBS 143366T | MF473311 | MF474161 | MF473734 |
| *Cladosporium* | *cladosporioides* | *Cladosporium vignae* | CBS 121.25T | HM148227 | HM148718 | HM148473 |
| *Cladosporium* | *cladosporioides* | *Cladosporium welwitschiicola* | CBS 142614T | KY646223 | KY646226 | KY646229 |
| *Cladosporium* | *cladosporioides* | *Cladosporium westerdijkiae* | CBS 113746T | HM148061 | HM148548 | HM148303 |
| *Cladosporium* | *cladosporioides* | *Cladosporium xanthochromaticum* | CBS 140691T | LN834415 | LN834599 | LN834511 |
| *Cladosporium* | *cladosporioides* | *Cladosporium xanthochromaticum* | DTO 323-E2 | MF473319 | MF474169 | MF473742 |
| *Cladosporium* | *cladosporioides* | *Cladosporium xanthochromaticum* | SFC20230103-M87 | OQ165104 | OQ185033 | OQ185114 |
| *Cladosporium* | *cladosporioides* | *Cladosporium xylophilum* | CBS 125997T | HM148230 | HM148721 | HM148476 |
| *Cladosporium* | *sphaerospermum* | *Cladosporium aciculare* | CBS 140488T | NR_152294 | KT600607 | KT600509 |
| *Cladosporium* | *sphaerospermum* | *Cladosporium aphidis* | CBS 132182T | NR_120010 | JN906997 | JN906984 |
| *Cladosporium* | *sphaerospermum* | *Cladosporium austrohemisphaericum* | CBS 140482T | KT600382 | KT600578 | KT600479 |
| *Cladosporium* | *sphaerospermum* | *Cladosporium coloradense* | CBS 143357T | NR_156347 | MF473795 | MF473372 |
| *Cladosporium* | *sphaerospermum* | *Cladosporium cycadicola* | CBS 137970T | NR_156279 | KJ869227 | KJ869236 |
| *Cladosporium* | *sphaerospermum* | *Cladosporium domesticum* | CBS 143358T | MF472955 | MF473805 | MF473382 |
| *Cladosporium* | *sphaerospermum* | *Cladosporium domesticum* | CPC 22402 | MF472959 | MF473809 | MF473386 |
| *Cladosporium* | *sphaerospermum* | *Cladosporium dominicanum* | CBS 119415T | NR_119603 | KJ596641 | JN906986 |
| *Cladosporium* | *sphaerospermum* | *Cladosporium dominicanum* | CPC 20109 | KT600391 | KT600586 | KT600488 |
| *Cladosporium* | *sphaerospermum* | *Cladosporium dominicanum* | EXF-696 | DQ780358 | EF101367 |  |
| *Cladosporium* | *sphaerospermum* | *Cladosporium dominicanum* | EXF-718 | DQ780356 | EF101370 |  |
| *Cladosporium* | *sphaerospermum* | *Cladosporium fusiforme* | CBS 119414T | NR_119608 | KJ596640 | JN906988 |
| *Cladosporium* | *sphaerospermum* | *Cladosporium fusiforme* | EXF-397 | DQ780389 | EF101373 | KJ596595 |
| *Cladosporium* | *sphaerospermum* | *Cladosporium fusiforme* | SFC102220 | MF186139 | MF185913 |  |
| *Cladosporium* | *sphaerospermum* | *Cladosporium halotolerans* | CBS 119416T | DQ780364 | KJ596633 | JN906989 |
| *Cladosporium* | *sphaerospermum* | *Cladosporium halotolerans* | CPC 22335 | MF472992 | MF473841 | MF473420 |
| *Cladosporium* | *sphaerospermum* | *Cladosporium herbarum* | CBS 121621T | EF679363 | EF679516 | EF679440 |
| *Cladosporium* | *sphaerospermum* | *Cladosporium langeronii* | CBS 189-54T | DQ780379 | EF101357 | JN906990 |
| *Cladosporium* | *sphaerospermum* | *Cladosporium lebrasiae* | CBS 138283T | KJ596568 | KJ596631 | KJ596583 |
| *Cladosporium* | *sphaerospermum* | *Cladosporium longissimum* | CBS 300-96T | DQ780352 | EF101385 | EU570259 |
| *Cladosporium* | *sphaerospermum* | *Cladosporium marinisedimentum* | MABIK_FU000+E2601143 | OQ186123 | OQ185171 | OQ185132 |
| *Cladosporium* | *sphaerospermum* | *Cladosporium marinisedimentum* | SFC20230103-M28 | OQ186124 | OQ185172 | OQ185133 |
| *Cladosporium* | *sphaerospermum* | *Cladosporium neolangeronii* | CBS 797-97T | MF473143 | MF473992 |  |
| *Cladosporium* | *sphaerospermum* | *Cladosporium neolangeronii* | CBS 109868 | DQ780377 | EF101362 | MF473571 |
| *Cladosporium* | *sphaerospermum* | *Cladosporium parahalotolerans* | CBS 139585T | KP701955 | KP702077 | KP701832 |
| *Cladosporium* | *sphaerospermum* | *Cladosporium parahalotolerans* | DTO 324-B7 | MF473169 | MF474017 | MF473592 |
| *Cladosporium* | *sphaerospermum* | *Cladosporium penidielloides* | CBS 140489T | NR_152295 | KT600608 | KT600510 |
| *Cladosporium* | *sphaerospermum* | *Cladosporium psychrotolerans* | CBS 119412T | KJ596573 | KJ596632 | KJ596610 |
| *Cladosporium* | *sphaerospermum* | *Cladosporium psychrotolerans* | EXF-332 | DQ780385 | EF101364 | KJ596591 |
| *Cladosporium* | *sphaerospermum* | *Cladosporium pulvericola* | CBS 143362T | MF473226 | MF474075 | MF473648 |
| *Cladosporium* | *sphaerospermum* | *Cladosporium salinae* | CBS 119413T | DQ780374 | EF101390 | JN906993 |
| *Cladosporium* | *sphaerospermum* | *Cladosporium salinae* | CBS 102047 | MF473251 | MF474101 | MF473674 |
| *Cladosporium* | *sphaerospermum* | *Cladosporium sloanii* | CBS 143364T | MF473253 | MF474103 | MF473676 |
| *Cladosporium* | *sphaerospermum* | *Cladosporium sphaerospermum* | CBS 193.54T | DQ780343 | EU570269 | EU570261 |
| *Cladosporium* | *sphaerospermum* | *Cladosporium sphaerospermum* | CBS 139576 | KP701884 | KP702007 | KP701761 |
| *Cladosporium* | *sphaerospermum* | *Cladosporium succulentum* | CBS 140466T | LN834434 | LN834618 | LN834530 |
| *Cladosporium* | *sphaerospermum* | *Cladosporium velox* | CBS 119417T | DQ780361 | EF101388 | JN906995 |
| *Cladosporium* | *sphaerospermum* | *Cladosporium velox* | CPC 22359T | MF473308 | MF474158 | MF473731 |
| *Cladosporium* | *sphaerospermum* | *Cladosporium velox* | EXF-466 | DQ780359 | EF101386 | KJ596597 |
| *Cladosporium* | *sphaerospermum* | *Cladosporium velox* | SFC20230103-M86 | OQ165103 | OQ185032 | OQ185113 |

***Fusarium incarnatum-equiseti* species complex**

| Species | Strain/Isolate | *CaM* | *RPB2* | *TEF1-α* |
| --- | --- | --- | --- | --- |
| *Fusarium aberrans* | CBS 119866 = MRC 6715 | MN170310 | MN170377 | MN170444 |
| *Fusarium aberrans* | CBS 131385^T^ | MN170311 | MN170378 | MN170445 |
| *Fusarium aberrans* | CBS 131387 | MN170312 | MN170379 | MN170446 |
| *Fusarium aberrans* | CBS 131388 | MN170313 | MN170380 | MN170447 |
| *Fusarium arcuatisporum* | NRRL 32997 | GQ505536 | GQ505802 | GQ505624 |
| *Fusarium brevicaudatum* | NRRL 43638^T^ | GQ505576 | GQ505843 | GQ505665 |
| *Fusarium brevicaudatum* | NRRL 43694 | GQ505579 | GQ505846 | GQ505668 |
| *Fusarium brevicaudatum* | NRRL 45998 | GQ505584 | GQ505851 | GQ505673 |
| *Fusarium bubalinum* | CBS 161.25 = NRRL 26857 = NRRL 26918^T^ | MN170314 | MN170381 | MN170448 |
| *Fusarium caatingaense* | CBS 976.97 | MN170315 | MN170382 | MN170449 |
| *Fusarium caatingaense* | NRRL 34003 = CBS 130317 | GQ505539 | GQ505805 | GQ505627 |
| *Fusarium camptoceras* | CBS 193.65 = ATCC 16065 = BBA 9810 = IMI 112500ET | MN170316 | MN170383 | MN170450 |
| *Fusarium cateniforme* | CBS 150.25 = ATCC 11853^T^ | MN170317 | MN170384 | MN170451 |
| *Fusarium caulicola* | GUCC 191051.1 = CGMCC 3.25475^T^ | OR043733 | OR043828 | OR043883 |
| *Fusarium caulicola* | GUCC 191051.2 | OR043734 | OR043829 | OR043884 |
| *Fusarium citri* | LC6896 = CGMCC 3.19467^T^ | MK289668 | MK289771 | MK289617 |
| *Fusarium citri* | NRRL 52765 = ARSEF 2304 | − | JF741165 | JF740839 |
| *Fusarium citrullicola* | SDBR-CMU422^T^ | OP020924 | OP020928 | OP020920 |
| *Fusarium citrullicola* | SDBR-CMU423 | OP020925 | OP020929 | OP020921 |
| *Fusarium clavum* | CBS 394.93 = BBA 64265 = NRRL 25795 | GQ505509 | GQ505775 | GQ505597 |
| *Fusarium clavum* | CBS 126202^T^ | MN170322 | MN170389 | MN170456 |
| *Fusarium clavum* | CBS 119881 = MRC 8412 | MN170323 | MN170390 | MN170457 |
| *Fusarium clavum* | NRRL 32871 = FRC R-9561 | GQ505531 | GQ505797 | GQ505619 |
| *Fusarium clavum* | NRRL 34032 | GQ505547 | GQ505813 | GQ505635 |
| *Fusarium clavum* | NRRL 34035 | GQ505549 | GQ505815 | GQ505637 |
| *Fusarium coffeatum* | CBS 635.76 = BBA 62053 = NRRL 20841^T^ | MN120696 | MN120736 | MN120755 |
| *Fusarium coffeatum* | NRRL 28577 = CBS 430.81 | MN120697 | MN120737 | MN120756 |
| *Fusarium compactum* | CBS 185.31 = NRRL 36318 | GQ505558 | GQ505824 | GQ505646 |
| *Fusarium compactum* | CBS 186.31 = NRRL 36323^ET^ | GQ505560 | GQ505826 | GQ505648 |
| *Fusarium croceum* | CBS 131777^T^ | MN170329 | MN170396 | MN170463 |
| *Fusarium croceum* | CBS 131788 | MN170330 | MN170397 | MN170464 |
| *Fusarium croceum* | CPC 35240 | MN170331 | MN170398 | MN170465 |
| *Fusarium croceum* | NRRL 3020 = FRC R-6053 = MRC 2231 | GQ505498 | GQ505764 | GQ505586 |
| *Fusarium croceum* | NRRL 3214 = FRC R-6054 = MRC 2232 | GQ505499 | GQ505765 | GQ505587 |
| *Fusarium duofalcatisporum* | CBS 264.50 = NRRL 36401 | GQ505563 | GQ505829 | GQ505651 |
| *Fusarium duofalcatisporum* | CBS 384.92 = NRRL 36448^T^ | GQ505564 | GQ505830 | GQ505652 |
| *Fusarium equiseti* | CBS 414.86 = FRC R-8508 = IMI 309348 | MN170333 | MN170400 | MN170467 |
| *Fusarium equiseti* | CBS 119663 | MN170334 | MN170401 | MN170468 |
| *Fusarium equiseti* | CPC 35220 | MN170337 | MN170404 | MN170471 |
| *Fusarium extenuatum* | LLC1492 | OP486038 | OP486727 | OP487157 |
| *Fusarium extenuatum* | LLC1501^T^ | OP486039 | OP486728 | OP487158 |
| *Fusarium fasciculatum* | CBS 131382^T^ | MN170339 | MN170406 | MN170473 |
| *Fusarium fasciculatum* | CBS 131383 | MN170340 | MN170407 | MN170474 |
| *Fusarium fasciculatum* | CBS 131384 | MN170341 | MN170408 | MN170475 |
| *Fusarium fecundum* | LC15875 = HSL1587 = CGMCC 3.23516^T^ | OQ125281 | OQ125544 | PV008845 |
| *Fusarium fecundum* | LC18376 = HSL197 | OQ125280 | OQ125543 | PV008846 |
| *Fusarium fecundum* | SAUCC 2414-4 = CGMCC 3.27792 | PQ309113 | PQ309121 | PV008845 |
| *Fusarium fici* | SAUCC 3249C-3 = CGMCC 3.27796 ^T^ | PQ309111 | PQ309123 | PQ309133 |
| *Fusarium flagelliforme* | CBS 162.57 = NRRL 36269^T^ | GQ505557 | GQ505823 | GQ505645 |
| *Fusarium flagelliforme* | CBS 259.54 = NRRL 36392 | GQ505562 | GQ505828 | GQ505650 |
| *Fusarium flagelliforme* | NRRL 26921 = CBS 731.87 | GQ505512 | GQ505778 | GQ505600 |
| *Fusarium flagelliforme* | NRRL 31011 = BBA 69079 | GQ505518 | GQ505784 | GQ505606 |
| *Fusarium gracilipes* | NRRL 43635^T^ | GQ505573 | GQ505840 | GQ505662 |
| *Fusarium guilinense* | NRRL 13335 = FRC R-2138 | GQ505502 | GQ505768 | GQ505590 |
| *Fusarium guilinense* | NRRL 32865 = FRC R-8480 | GQ505526 | GQ505792 | GQ505614 |
| *Fusarium hainanense* | NRRL 26417 = CBS 544.96 | GQ505510 | GQ505776 | GQ505598 |
| *Fusarium humuli* | LC 4490 | MK289664 | MK289767 | MK289614 |
| *Fusarium humuli* | LC 12158 | MK289645 | MK289745 | MK289592 |
| *Fusarium humuli* | LC 12159 | MK289646 | MK289746 | MK289593 |
| *Fusarium incarnatum* | NRRL 32866 = FRC R-8822 | GQ505527 | GQ505793 | GQ505615 |
| *Fusarium ipomoeae* | CBS 135762 | MN170344 | MN170411 | MN170478 |
| *Fusarium ipomoeae* | CBS 140909 | MN170345 | MN170412 | MN170479 |
| *Fusarium ipomoeae* | NRRL 34039 | GQ505551 | GQ505817 | GQ505639 |
| *Fusarium irregulare* | NRRL 31160 | GQ505519 | GQ505785 | GQ505607 |
| *Fusarium irregulare* | NRRL 32182 | GQ505523 | GQ505789 | GQ505611 |
| *Fusarium irregulare* | NRRL 32869 = FRC R-9445 | GQ505530 | GQ505796 | GQ505618 |
| *Fusarium jinanense* | LC15878 = HSL751 = CGMCC 3.23519^T^ | OQ125271 | OQ125521 | OQ125131 |
| *Fusarium jinanense* | LC18379 = HSL1983 | OQ125272 | OQ125522 | OQ125132 |
| *Fusarium kotabaruense* | InaCC F963^T^ | LS479429 | LS479859 | LS479445 |
| *Fusarium lacertarum* | NRRL 20423 = ATCC 42771 = CBS 130185 = IMI 300797T | GQ505505 | GQ505771 | GQ505593 |
| *Fusarium lacertarum* | NRRL 36123 = CBS 102300 | GQ505555 | GQ505821 | GQ505643 |
| *Fusarium longicaudatum* | CBS 123.73 = ATCC 24370 = IMI 160825 = NRRL 25477T | MN170347 | MN170414 | MN170481 |
| *Fusarium longifundum* | CBS 235.79 = NRRL 36372^T^ | GQ505561 | GQ505827 | GQ505649 |
| *Fusarium luffae* | CBS 131097 | MN170348 | MN170415 | MN170482 |
| *Fusarium luffae* | NRRL 31167 | GQ505520 | GQ505786 | GQ505608 |
| *Fusarium luffae* | NRRL 32522 | GQ505524 | GQ505790 | GQ505612 |
| *Fusarium mianyangense* | LC15879 = HSL859 = CGMCC 3.23520^T^ | OQ125335 | OQ125510 | OQ125232 |
| *Fusarium monophialidicum* | NRRL 54973 | MN170349 | MN170416 | MN170483 |
| *Fusarium mucidum* | CBS 102394 | MN170350 | MN170417 | MN170484 |
| *Fusarium mucidum* | CBS 102395^T^ | MN170351 | MN170418 | MN170485 |
| *Fusarium mucidum* | Indo 175 | LS479431 | LS479862 | LS479447 |
| *Fusarium multiceps* | CBS 130386 = NRRL 43639^T^ | GQ505577 | GQ505844 | GQ505666 |
| *Fusarium nanum* | CBS 119867 = FRC R-4237 = MRC 3228 | MN170352 | MN170419 | MN170486 |
| *Fusarium nanum* | CBS 131781 | MN170353 | MN170420 | MN170487 |
| *Fusarium nanum* | NRRL 32993 | GQ505532 | GQ505798 | GQ505620 |
| *Fusarium neoscirpi* | CBS 610.95 = NRRL 26861 = NRRL 26922^T^ | GQ505513 | GQ505779 | GQ505601 |
| *Fusarium neosemitectum* | CBS 189.60^T^ | MN170355 | MN170422 | MN170489 |
| *Fusarium neosemitectum* | CBS 190.60 | MN170356 | MN170423 | MN170490 |
| *Fusarium nothincarnatum* | LC18382 = HSL199 | OQ125289 | OQ125508 | OQ125146 |
| *Fusarium nothincarnatum* | LC18436 = HSL221 = CGMCC 3.24286^T^ | OQ125290 | OQ125509 | OQ125147 |
| *Fusarium pernambucanum* | CBS 132194 | MN170358 | MN170425 | MN170492 |
| *Fusarium pernambucanum* | CBS 133024 | MN170360 | MN170427 | MN170494 |
| *Fusarium persicinum* | CBS 479.83^T^ | MN170361 | MN170428 | MN170495 |
| *Fusarium persicinum* | CBS 143596 = CPC 30848 | LT970732 | LT970751 | LT970779 |
| *Fusarium persicinum* | CBS 143598 = CPC 30850 | LT970733 | LT970752 | LT970780 |
| *Fusarium persicinum* | CBS 143600 = CPC 30852 | LT970734 | LT970753 | LT970781 |
| *Fusarium persicinum* | CBS 143606 = CPC 30858 | LT970736 | LT970755 | LT970783 |
| *Fusarium radicigenum* | GUCC 197371.1 | OR043752 | OR043851 | OR043907 |
| *Fusarium radicigenum* | GUCC 197425.1 | OR043753 | OR043852 | OR043908 |
| *Fusarium radicigenum* | GUCC 197221.1 = CGMCC 3.25478^T^ | OR043754 | – | OR043909 |
| *Fusarium rhinolophicola* | KUMCC 21-0449^T^ | OR022061 | OR025917 | OR026001 |
| *Fusarium rhinolophicola* | KUMCC 21-0450 | OR022063 | OR025919 | OR026003 |
| *Fusarium scirpi* | CBS 447.84 = FRC R-6252 = NRRL 36478^NT^ | GQ505566 | GQ505832 | GQ505654 |
| *Fusarium scirpi* | CBS 448.84 = FRC R-6253 | MN170364 | MN170431 | MN170498 |
| *Fusarium scirpi* | NRRL 13402 | GQ505504 | GQ505770 | GQ505592 |
| *Fusarium serpentinum* | CBS 119880 = BBA 62209 = MRC 1813 | MN170365 | MN170432 | MN170499 |
| *Fusarium sulawesiense* | InaCC F940^T^ | LS479422 | LS479855 | LS479443 |
| *Fusarium sulawesiense* | InaCC F941 | LS479423 | LS479856 | LS479444 |
| *Fusarium tanahbumbuense* | CBS 145.44 = BBA 4095 | MN170371 | MN170438 | MN170505 |
| *Fusarium tanahbumbuense* | CBS 131009 | MN170372 | MN170439 | MN170506 |
| *Fusarium tanahbumbuense* | InaCC F965^T^ | LS479432 | LS479863 | LS479448 |
| *Fusarium tanahbumbuense* | NRRL 34005 | GQ505541 | GQ505807 | GQ505629 |
| *Fusarium tanahbumbuense* | NRRL 43297 | GQ505569 | GQ505835 | GQ505657 |
| *Fusarium tangerinum* | LLC3018 | OP486066.1 | OP486757.1 | OP487188.1 |
| *Fusarium tangerinum* | LLC3501^T^ | OP486067.1 | OP486758.1 | OP487189.1 |
| *Fusarium toxicum* | CBS 219.63 | MN170373 | MN170440 | MN170507 |
| *Fusarium toxicum* | CBS 406.86 = FRC R-8507 = IMI 309347 = NRRL 25796T | MN170374 | MN170441 | MN170508 |
| *Fusarium toxicum* | CBS 130385 | MN170375 | MN170442 | MN170509 |
| *Fusarium toxicum* | NRRL 43636 | GQ505574 | GQ505841 | GQ505663 |
| *Fusarium weifangense* | LC18333 = HSL1800 = CGMCC 3.24285^T^ | OQ125276 | OQ125515 | OQ125107 |
| *Fusarium weifangense* | LC18243 = HSL102 | OQ125273 | OQ125513 | OQ125106 |
| *Fusarium weifangense* | SAUCC 5208C-2 = CGMCC 3.27939 | PQ309117 | PQ309125 | PQ309127 |
| *Fusarium weifangense* | GUCC 191050.1 = CGMCC 3.25474 | OR043731 | OR043826 | OR043881 |
| *Fusarium weifangense* | GUCC 191050.2 | OR043732 | OR043827 | OR043882 |
| *Fusarium wereldwijsianum* | CBS 148219 = NL19-99002 | MZ921536 | MZ921716 | MZ921848 |
| *Fusarium wereldwijsianum* | CBS 148220 = NL19-99003 | MZ921537 | MZ921717 | MZ921849 |
| *Fusarium xylosmatis* | SAUCC 2416-1 = CGMCC 3.27794^T^ | PQ309115 | PQ309119 | PQ309131 |
| *Fusarium khuzestanicum* | IRAN 4863C | PP858502 | PP858506 | PP858510 |
| Fusarium oryzicola | IRAN4864C | PP858501 | PP858505 | PP858509 |
