# Supplementary figures and images for: Spider webs as reservoirs of culturable fungal diversity: evidence from orb-weaving Cyclosa mulmeinensis spider in Thai rice agroecosystems
Source: Biodivers Data J. 2026 Apr 20;14:e187035. doi: 10.3897/BDJ.14.e187035 (PMC13122186; doi:10.3897/BDJ.14.e187035)

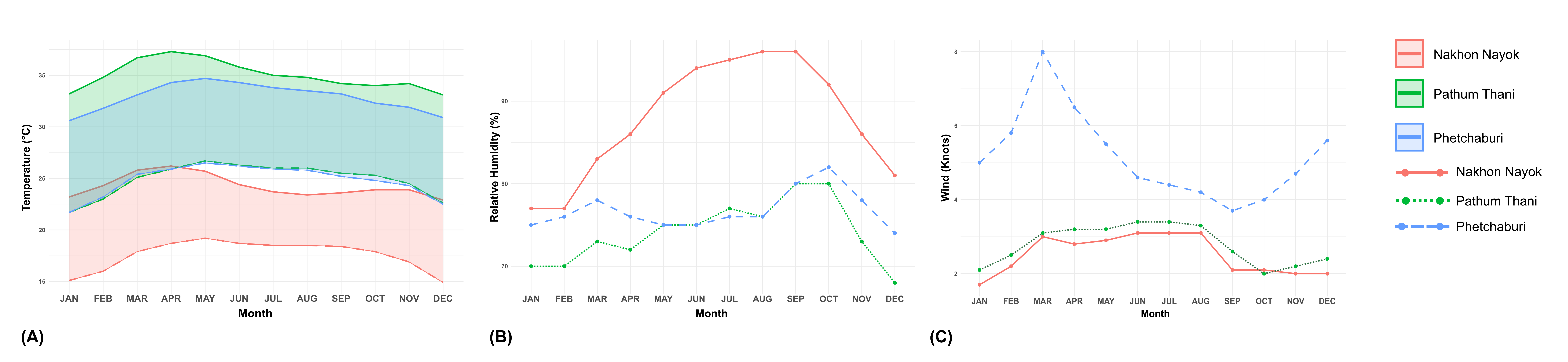

Supplement: Supplementary material 5 — Weather data (2014–2023) [file bdj-14-e187035-s005.png]

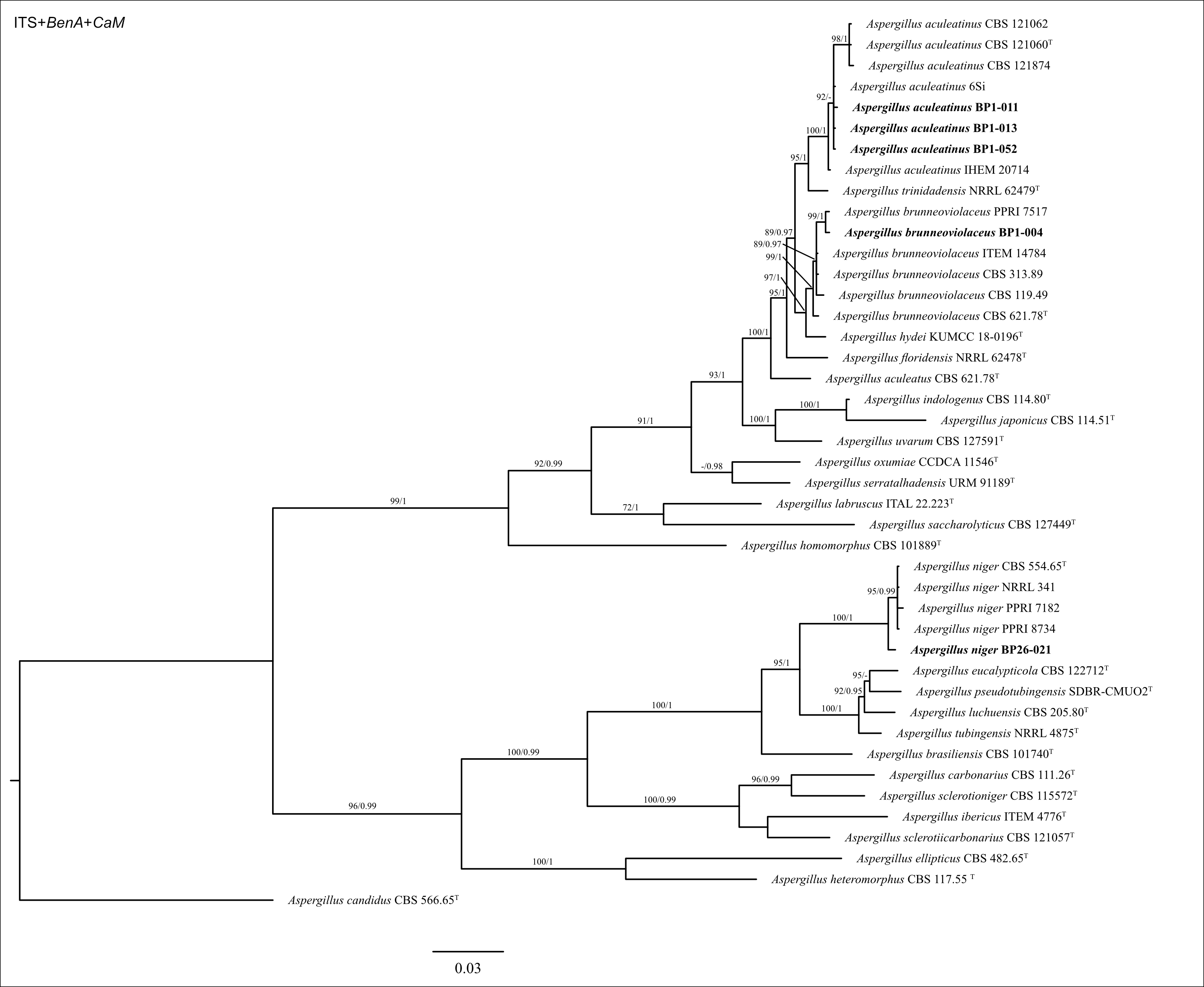

Supplement: Supplementary material 6 — Maximum Likelihood phylogenetic tree, representing Aspergillus section Nigri [file bdj-14-e187035-s006.png]

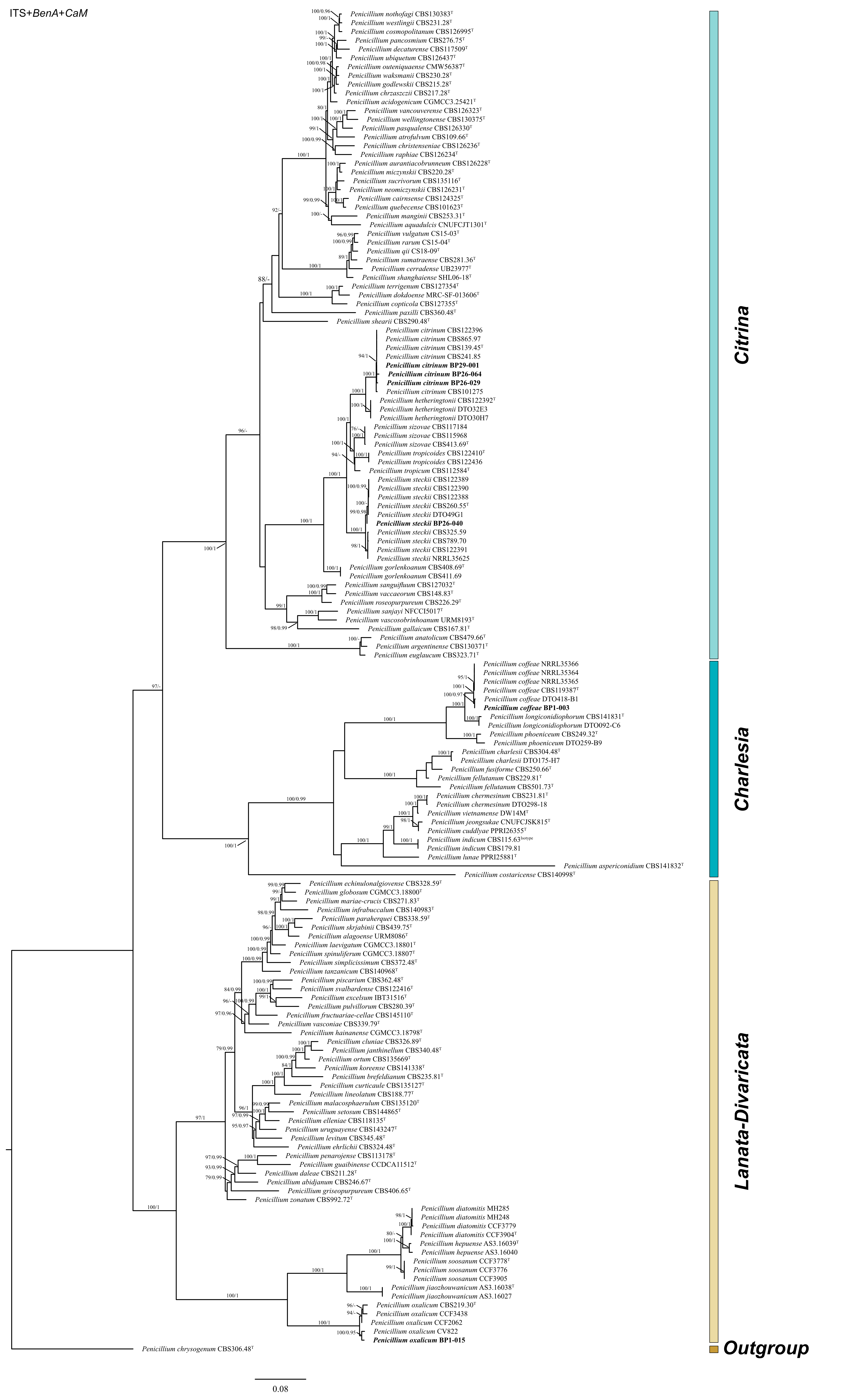

Supplement: Supplementary material 7 — Maximum Likelihood phylogenetic tree, representing Penicillium section Citrina [file bdj-14-e187035-s007.png]

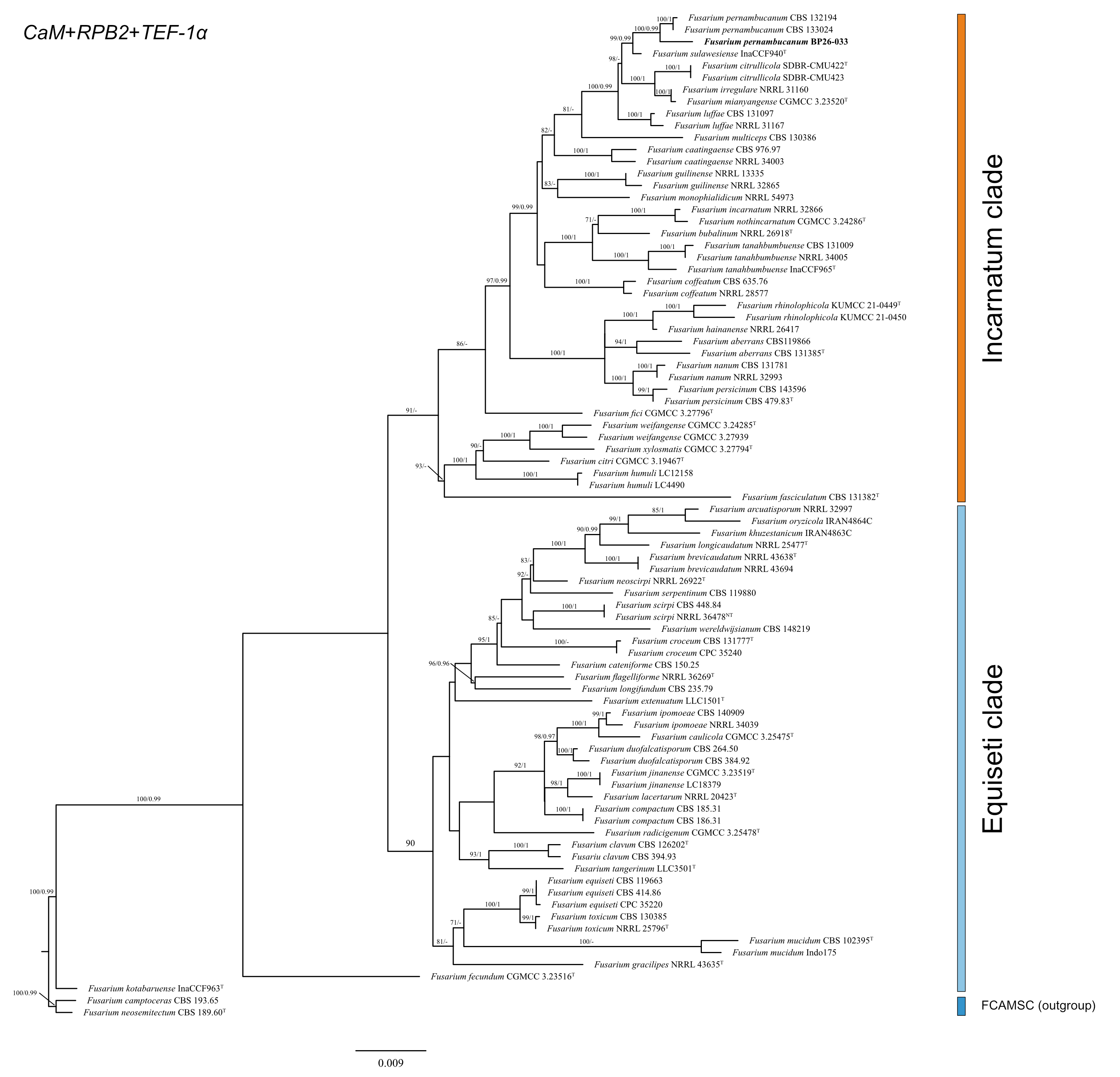

Supplement: Supplementary material 9 — Maximum Likelihood phylogenetic tree, representing Fusarium incaarnatum-equiseti species complex [file bdj-14-e187035-s009.png]

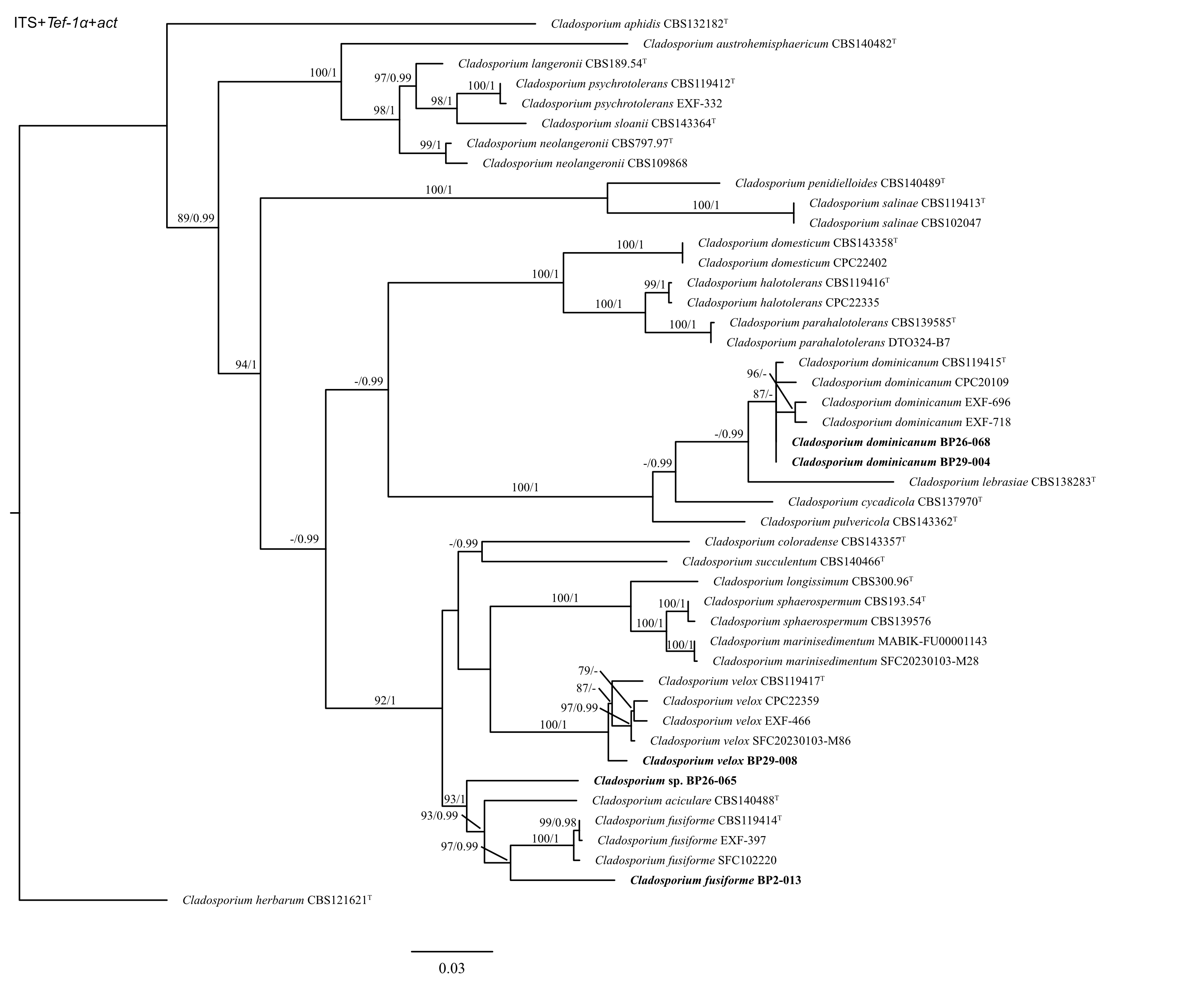

Supplement: Supplementary material 11 — Maximum Likelihood phylogenetic tree, representing Cladosporium sphaerospermum species complex [file bdj-14-e187035-s011.png]

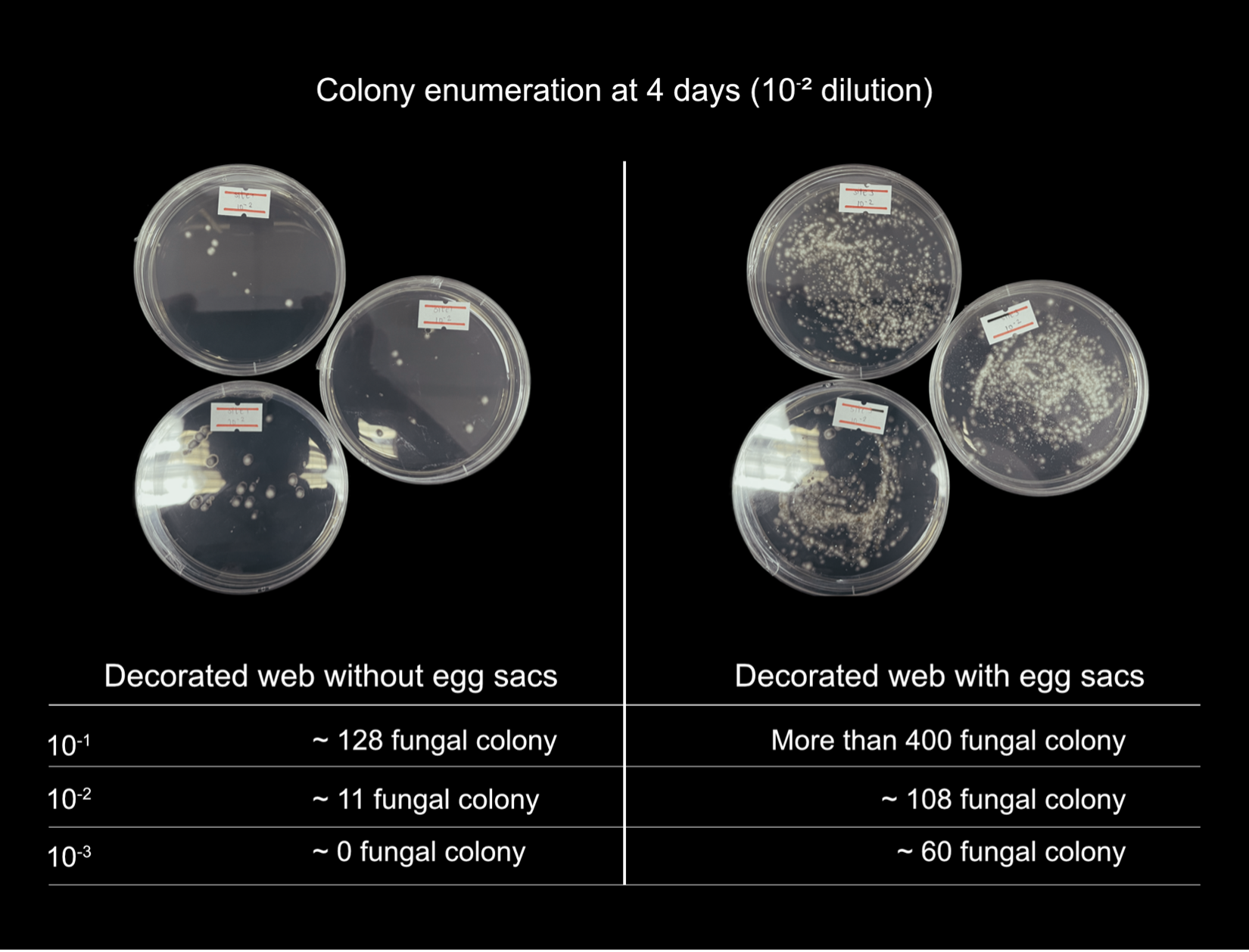

Supplement: Supplementary material 12 — PDA plate cultures [file bdj-14-e187035-s012.png]
